# Plasma proteomic analysis to identify potential biomarkers of histologic chorioamnionitis in women with preterm premature rupture of membranes

**DOI:** 10.1371/journal.pone.0270884

**Published:** 2022-07-07

**Authors:** Ji Eun Lee, Kisoon Dan, Hyeon Ji Kim, Yu Mi Kim, Kyo Hoon Park

**Affiliations:** 1 Center for Theragnosis, Biomedical Research Division, Korea Institute of Science and Technology, Seoul, Korea; 2 Proteomics Core Facility, Biomedical Research Institute, Seoul National University Hospital, Seoul, Korea; 3 Departments of Obstetrics and Gynecology, Seoul National University College of Medicine, Seoul National University Bundang Hospital, Seongnam, Korea; University of Copenhagen: Kobenhavns Universitet, DENMARK

## Abstract

**Introduction:**

To identify potential biomarkers in the plasma that could predict histologic chorioamnionitis (HCA) in women with preterm premature rupture of membranes (PPROM), using shotgun and targeted proteomic analyses.

**Methods:**

This retrospective cohort study included 78 singleton pregnant women with PPROM (24–34 gestational weeks) who delivered within 96 h of blood sampling. Maternal plasma samples were analyzed by label-free liquid chromatography-tandem mass spectrometry for proteome profiling in a nested case-control study design (HCA cases vs. non-HCA controls [n = 9 each]). Differential expression of 12 candidate proteins was assessed by multiple reaction monitoring-mass spectrometry (MRM-MS) analysis in individual plasma samples from cases and controls matched by gestational age at sampling (n = 40, cohort 1). A validation study was further performed in an independent study group (n = 38, cohort 2) using ELISA and turbidimetric immunoassay for three differentially expressed proteins.

**Results:**

Shotgun proteomics analyses yielded 18 proteins that were differentially expressed (*P* < 0.05) between HCA cases and non-HCA controls. MRM-MS analysis of 12 differentially expressed proteins further revealed that the CRP, C4A, and SAA4 levels were significantly increased in women with HCA. A multi-marker panel comprising plasma SAA4 and C4A showed enhanced potential for differentiating HCA from non-HCA women (**area under the curve =** 0.899). Additional validation of these findings by ELISA assays revealed that the CRP levels were significantly higher in women with HCA than in those without HCA, whereas the plasma levels of C4A and SAA4 did not significantly differ between the two groups.

**Conclusions:**

Plasma C4A, SAA4, and CRP were identified as potential biomarkers for detecting HCA in women with PPROM, based on targeted and shotgun proteomic analyses, showing good accuracy when used as a combined dual-biomarker panel (C4A and SAA4). Nevertheless, ELISA validation of these proteins, except for CRP, may not yield clinically useful markers for predicting HCA.

## Introduction

Preterm premature rupture of membranes (PPROM), which precedes approximately 30–40% of preterm births, is the main cause of neonatal morbidity, mortality, and long-term sequelae [1, 2]. In particular, almost half of all PPROM cases are frequently complicated by subclinical acute inflammation in the placenta and fetal tissue, commonly named as acute histologic chorioamnionitis [HCA] [3, 4]. Increasing evidences suggest that HCA carries additional risks to both the pregnant women and their fetuses, including greater risk of imminent preterm birth, as well as sepsis, neurologic morbidity, and mortality in neonates [5–9]. However, the diagnosis of HCA is possible only after delivery, and hence, cannot be implemented in clinical practice. Thus, more accurate and early prenatal predictive markers (especially noninvasive ones) are urgently needed for identifying subclinical HCA in the context of PPROM.

Traditionally, inflammatory biomarkers in the amniotic fluid (AF) detected via amniocentesis, including pro-inflammatory cytokines and matrix metalloproteinases (MMPs), have been considered to be clinically useful predictors of subclinical HCA in women with PPROM [6, 7, 10, 11]. However, amniocentesis is invasive and technically difficult to perform, especially in women with severe oligohydramnios secondary to PPROM, thereby limiting the utility of these markers in the clinical practice. Importantly, increased levels of various cytokines and MMPs were reported to occur concurrently in the maternal blood and AF compartments in cases of HCA/microbial invasion of the amniotic cavity (MIAC), in the context of PPROM [12–14]. Moreover, as a hallmark of inflammation of the chorioamniotic membranes, neutrophils derived from the fetal membranes in acute HCA are predominantly of maternal origin, whereas the neutrophils found in the umbilical cord and chorionic vessels on the chorionic plate of placenta are of fetal origin [15–17]. Fetal cells and fetal exosomes enter the maternal circulation during pregnancy [18, 19]. Thus, the development of acute HCA may be well reflected in the maternal circulation via mediators associated with neutrophils derived from maternal and fetal origin. Consequently, an approach using maternal blood samples may provide a feasible alternative to AF assessment for the prediction of HCA in PPROM. However, to date, few information is available on the role of multiple protein mediators in maternal circulation that are causally linked to HCA development in women with PPROM, particularly when assessed using high-throughput screening platforms.

Noteworthy, quantitative mass spectrometry (MS)-based proteomics has recently emerged as one of the most powerful tools for high-throughput protein screening, particularly for disease-specific proteins of low abundance [20], showing promising results for the discovery of novel serum protein markers in the field of complex syndromes with multiple causes, such as preterm birth [21–23]. In particular, multiple reaction monitoring-mass spectrometry (MRM-MS) analysis became the primary technique for quantitative proteomics, which provides high selectivity, sensitivity, and the capability to quantitate numerous peptides or proteins simultaneously [24, 25]. However, to date, the use of the aforementioned approaches to identify biomarkers of HCA in maternal blood from women with PPROM has not been attempted, despite that this approach in the discovery phase has been applied using PPROM AF samples in relation to acute HCA [26–28]. We hypothesized that shotgun and targeted proteomic analyses of the plasma samples would lead to the identification of a set of sensitive, novel markers of acute HCA among patients with PPROM. Our aim was to identify potential plasma biomarkers that could predict HCA in women with PPROM, using shotgun and targeted proteomic analyses.

## Materials and methods

### Study design and participants

A retrospective cohort study was conducted in women with singleton pregnancies who were admitted to the Department of Obstetrics and Gynecology at the Seoul National University Bundang Hospital, Seongnamsi, Republic of Korea, between June 2004 and July 2015. Women who were diagnosed with PPROM from 24+0 to 34+6 weeks of gestation, who delivered a live fetus within four days (96 h) of blood sample collection, and had available data for placental histopathology were included in the study. Women were excluded if they had (1) a time interval of more than 96 h from plasma sampling to delivery (this criterion was used to preserve a significant time-related association between the proteins assayed in the blood and the occurrence of pathological placental lesions), (2) multiple gestations, (3) evidence of clinical chorioamnionitis at the time of admission; and (4) a fetus with major congenital anomalies. PPROM was defined as clinically confirmed fetal membrane rupture with leakage of AF preceding the onset of labor and at < 37 weeks of gestation. This condition was diagnosed by visual examination, using a sterile speculum, to confirm pooling of AF in the vagina (or fluid leakage from the cervix) in association with a positive nitrazine test and/or AmniSure ROM test (Qiagen, Hilden, Germany). The primary outcome measure was subclinical histologic chorioamnionitis. The study was approved by the Ethics Committee of the Seoul National University Bundang Hospital (project number B-1311/228-010). Written informed consent was obtained from all study participants for the collection and use of blood samples and the use of the clinical information for research purposes.

**Fig 1:**
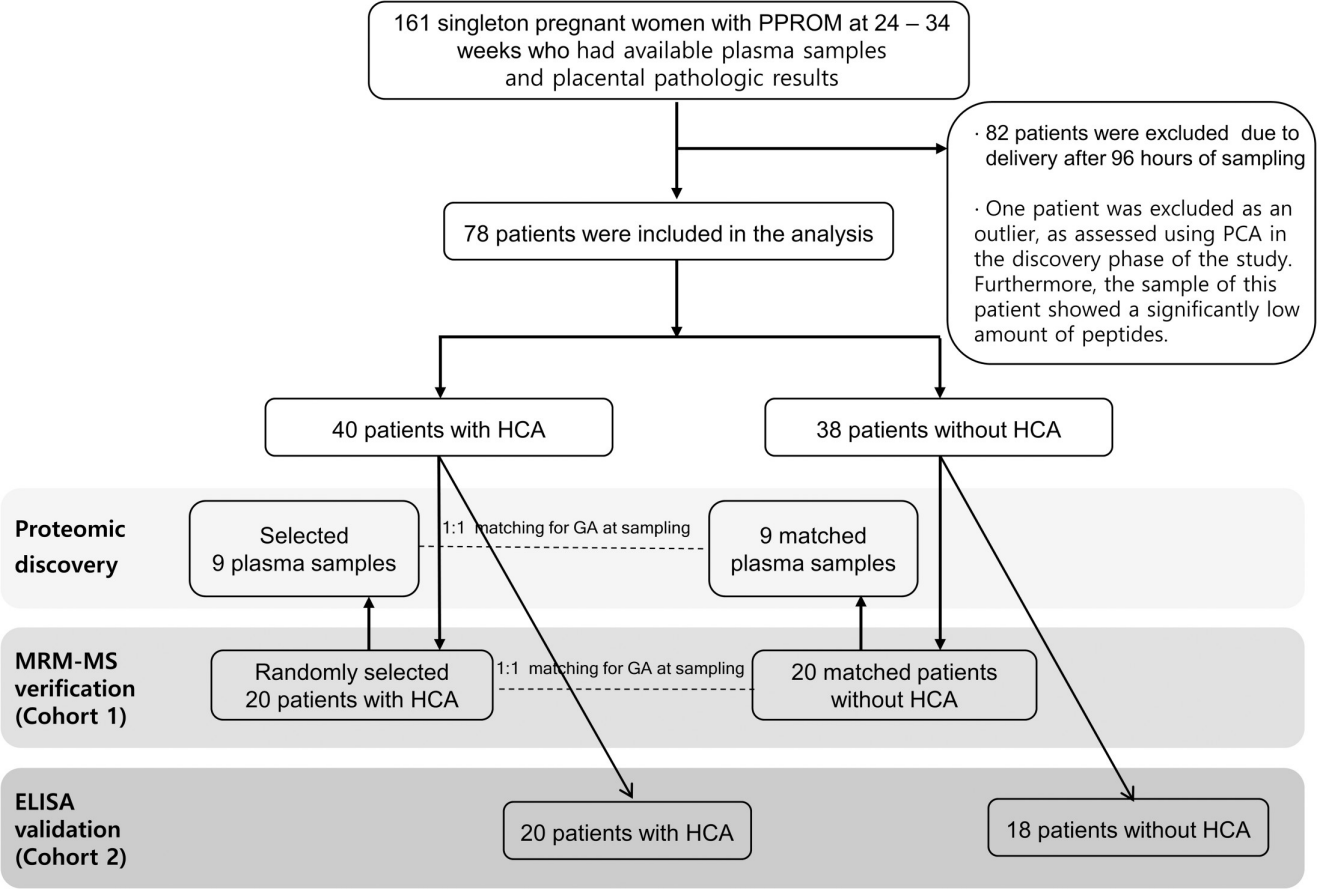
A brief flow chart of patients included in the proteomic and immunoassay analyses. ELISA, enzyme-linked immunosorbent assay; HCA, histologic chorioamnionitis; PPROM, preterm premature rupture of membranes; MRM-MS, multiple reaction monitoring-mass spectrometry.

Fig 1 shows a brief flowchart of the inclusion of the participants according to the experimental phases. For the proteomic study based on label-free liquid chromatography (LC)-tandem mass spectrometry (MS/MS) and verification using MRM-MS analysis, 20 patients with HCA (case subjects) were randomly selected among the initial 40 HCA patients using a random sequence generator and 20 control patients were matched according to parity, length of specimen storage (± 5 years), maternal age (± 5 years), and gestational age at sampling (± 1.5 weeks) (n = 40, cohort 1). In the discovery phase using label-free LC-MS/MS, a nested case-control study comprising 9 patients with PPROM and HCA (case subjects) and 9 gestational age-matched patients with PPROM without HCA (control subjects) was conducted, all of which were selected among cohort 1 (Fig 1). To verify the biomarker candidates selected from the discovery experiment, MRM-MS was performed in the MRM-verification set of 40 individual samples (cohort 1). Finally, in the enzyme-linked immunosorbent assays (ELISA) validation phase, the levels of transforming growth factor-beta-induced (TGFBI), which was accidentally missed in the MRM analysis, and biomarkers showing significant association with HCA in the MRM-MS verification phase were assessed using plasma samples from the remaining 20 HCA cases and 18 non-HCA controls (n = 38, cohort 2) (Fig 1).

### Biological sample collection and processing

At enrollment, maternal venous blood samples were collected in ethylenediaminetetraacetic acid tubes after routine measurements of serum C-reactive protein (CRP) concentration in patients diagnosed with PPROM. The CRP concentrations were measured using a latex-enhanced turbidimetric immunoassay (Denka Seiken, Tokyo, Japan) in an automated analyzer (Toshiba 200FR; Toshiba, Tokyo, Japan). The samples were immediately centrifuged at 1500 × *g* for 10 min, and the supernatant was aliquoted and stored at −70°C until further use.

The methods for the collection of placental tissue samples and processing for histologic assessment have previously been reported [29, 30], and a detailed description is provided in Supplementary Materials. Placental histopathology was performed by board-certified pathologists blinded to the clinical data.

### Definition, diagnosis, and management of PPROM

Management of PPROM and pathological diagnosis of acute and clinical chorioamnionitis were conducted as previously described [31–33], and detailed descriptions are provided in Supplementary Materials. Briefly, prophylactic broad-spectrum antibiotics (ampicillin plus macrolides [azithromycin, clarithromycin, or erythromycin]) were administered to all patients presenting with PPROM. For patients with PPROM pregnancies between 23 and 34 weeks of gestation, antenatal corticosteroids were used to support fetal lung maturation. Intravenous tocolytic therapy, such as atosiban, magnesium sulfate, or ritodrine, was administered to patients with PPROM at < 34 weeks of gestation at the discretion of the attending physicians.

### Diagnosis of HCA and funisitis

Acute HCA was diagnosed when acute inflammatory change was detected in any tissue sample (fetal membranes [amnion and chorion-decidua], chorionic plate, or umbilical cord), in accordance with a previously detailed definition [30]. Acute funisitis was diagnosed when neutrophils infiltrated the wall of the umbilical cord vessels and/or Wharton’s jelly using previously published criteria. The presence and degree of acute inflammation was assessed and classified as grade 0, 1, or 2 based on previously published criteria [30].

### Label-free LC-MS/MS analysis (discovery phase)

Individual plasma samples from HCA cases (n = 9) and non-HCA controls (n = 9) were used to identify potential plasma protein biomarker candidates. Proteins from each plasma sample were digested with trypsin and analyzed using a Q Exactive Plus Hybrid Quadrupole-Orbitrap Mass Spectrometer (Thermo Fisher Scientific, Waltham, MA, USA) coupled to an Ultimate 3000 RSLC system (Dionex, Waltham, MA, USA) with a nanoelectrospray source. The MS and MS/MS data were searched against the UniProt human database (release date: December 2014) for protein identification using MaxQuant (version 1.6.1.0.) [34, 35]. To extract differentially expressed proteins (DEPs) between non-HCA and HCA groups, bioinformatics analysis was performed using Perseus software (version 1.6.0.2, https://maxquant.net/perseus/) [36, 37]. Detailed descriptions of the discovery phase experiments are provided in Supplementary Materials. The spectra generated in this study has been submitted to the PRIDE database; Project Accession PXD027573 (to view please use reviewer account username: reviewer_pxd027573@ebi.ac.uk and password: HFm1lofE).

### Targeted LC-MRM-MS analysis (verification phase)

Target proteins selected from DEPs acquired by label-free quantification were subjected to LC-MRM-MS analyses using a triple quadrupole (QQQ) mass spectrometer to measure and compare the abundance of the target proteins in individual samples (detailed description of MRM method development is provided in Supplementary Materials). For MRM verification, 12 proteins, except for five immunoglobulin-domain proteins and (accidentally missed) TGFBI, were selected for the MRM-MS assay. A multiplexed MRM method that quantified 49 surrogate peptides derived from 12 proteins in a one-time sample injection was established. Relative quantification of each target peptide was performed using 40 individual plasma samples from cohort 1 and the established LC-MRM-MS method. The MRM data are deposited into Panorama Public: https://panoramaweb.org/0EOuoM.url (to view please use reviewer account username: panorama+reviewer54@proteinms.ne and password: zhSUQMpm).

### Enzyme-linked immunosorbent assays (ELISA) (validation phase)

Based on the proteomics results, the plasma concentration of complement C4-A (C4A; BD Biosciences, San Jose, CA, USA), serum amyloid A4 (SAA4; Novus Biologicals, Centennial, CO, USA), and TGFBI (DuoSet ELISA; R&D System, Minneapolis, MN, USA) were further validated in 38 individual samples (cohort 2) using ELISA kits, according to the manufacturers’ instructions. Prior to the measurement of the protein levels, the maternal plasma samples were diluted at 1:4 for SAA4, 1:5000 for TGFBI, and 1:100000 for C4A. For the SAA4 assay, the plasma samples that contained SAA4 at concentrations higher than 20.0 ng/mL were diluted at 1:30 for final analysis. The intra- and interassay coefficients of variation were 4.1% and 6.6% for C4A, 4.1% and 10.2% for SAA4, and 1.6% and 4.3% for TGFBI, respectively.

### Statistical analysis

Data analyses were performed using SPSS version 25.0 (IBM Corp., Armonk, NY, USA). Comparison of clinical data, relative abundance levels of the candidate proteins, and their concentrations obtained by ELISA was performed using the Mann-Whitney *U* test for non-parametric variables. Comparison of categorical data was performed using the chi-squared or Fisher’s exact tests, where appropriate. MRM results were expressed as mean ± standard deviation and were further analyzed using multivariate logistic regression analyses to evaluate the independent relationship of the plasma levels of the candidate biomarkers with HCA risk, adjusting for baseline covariates (such as gestational age at sampling) that had a *P*-value < 0.05 in univariate analysis. In the logistic regression model, continuous data of various biomarkers were transformed into binary variables defined by cutoff values, according to their receiver operating characteristics (ROC) curve, to overcome the imposed analytical limitations of their left-skewed distribution and achieve clinically relevant decisions [38]. The best cutoff points were determined using the maximum Youden index (maximum [sensitivity + specificity– 1]). Finally, to establish the best protein panel to distinguish patients with and without HCA based on the identified candidate plasma biomarkers, a multivariate logistic regression analysis was performed using the forward stepwise method. Prior to the construction of a multi-marker panel, we performed correlation analyses between all markers to evaluate multicollinearity among the 11 candidate peptides that were significantly up- or down-regulated, as demonstrated by MRM-MS analyses. The peak area ratios (PARs) of different peptides derived from the same protein showed almost perfect correlation with each other (four peptides for CRP: r = 0.891–0.996; five peptides for the SAA4 protein: r = 0.701–0.988; and two peptides for the C4A protein: r = 0.861). Hence, in this stepwise regression model, only one peptide per protein, which was selected based on the highest value of AUCs of different peptides, was included. The Spearman’s rank correlation test was used to evaluate the correlation between the PARs of the markers. The dichotomized variables were also used as independent variables to develop a plasma multiple-biomarker panel. Using a previously described method [39], the areas under the ROC curves (AUCs) for each protein were calculated and compared. All probability values indicated are two-tailed, and *P*-values < 0.05 were deemed statistically significant.

## Results

During the study period, 161 consecutive women diagnosed with PPROM who met the inclusion criteria were recruited for this study, among whom 82 delivered after 96 h of sampling; thus were excluded from the study (Fig 1). During the biomarker discovery phase, one patient was further excluded because her plasma sample was identified as an outlier by principal component analysis (PCA; S1 Fig) and because it showed a remarkably low amount of peptides. The amount of peptides in the outlier ranged from 23.3% to 36.8% compared to that in the remaining samples. Overall, 78 samples from women with PPROM (40 cases of HCA and 38 controls without HCA) were used for the present analyses. A detailed description of the inclusion of study participants employed for proteomic studies in the discovery and verification stages has been provided in the Supplementary Materials.

### Demographic and clinical characteristics of the discovery cohorts

The baseline clinical characteristics of the discovery cohort used for quantitative label-free proteomic analysis for the identification of biomarkers associated with HCA are described in Supplementary Information S1 Table. Owing to matching, HCA cases and non-HCA controls did not differ regarding gestational age at sampling, parity, maternal age, and medication use.

### Quantitative proteomic analysis and selection of DEPs (discovery phase)

**Fig 2:**
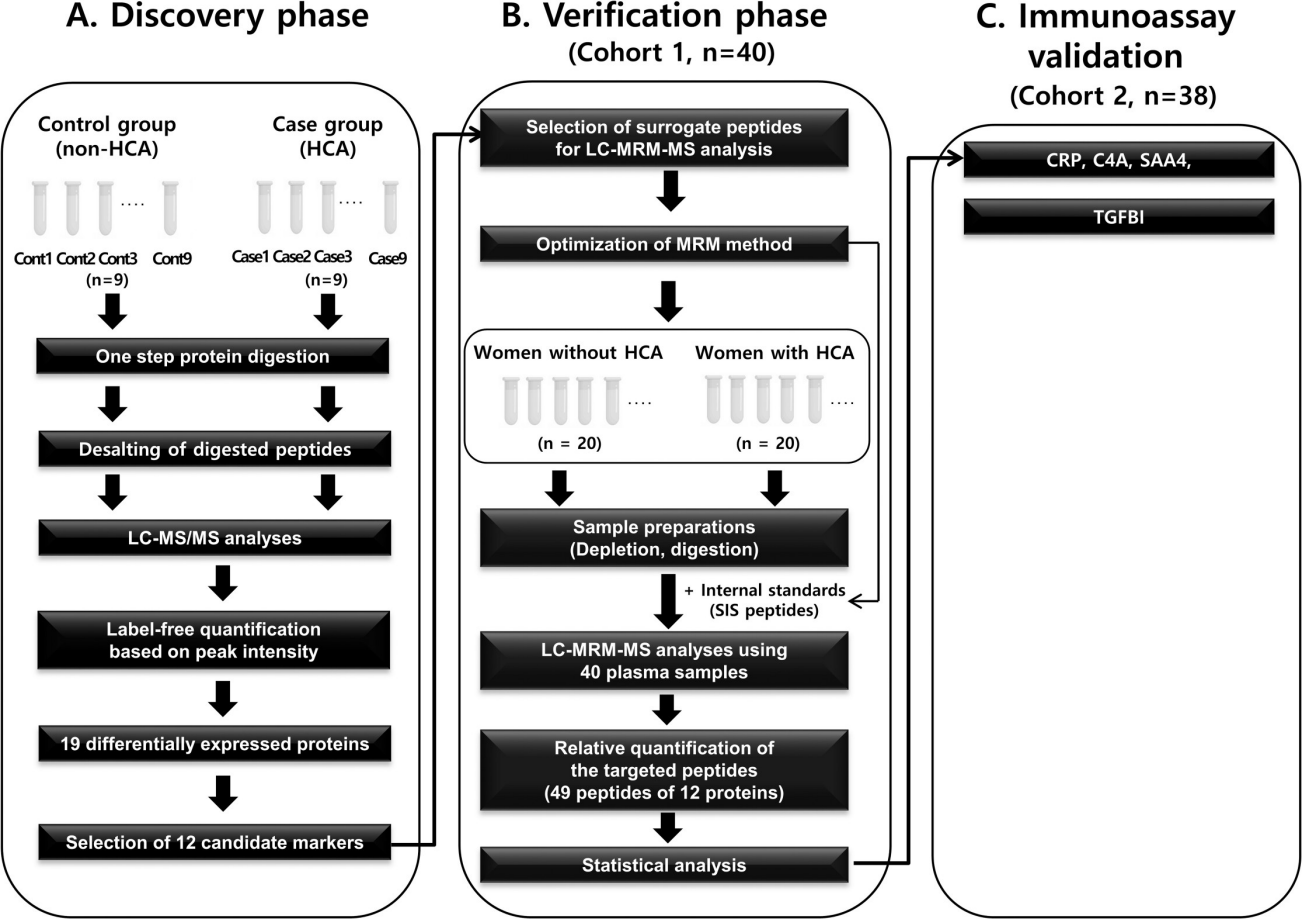
Schematic workflow of the discovery (label-free LC-MS/MS), verification (LC-MRM-MS), and validation (immunoassay) experiments. C4A, complement C4-A; CRP, C-reactive protein; HCA, histologic chorioamnionitis; LC, liquid chromatography; MRM-MS, multiple reaction monitoring-mass spectrometry; MS/MS, tandem mass spectrometry; SAA4; serum amyloid A4; SIS, stable isotope-labeled standard; TGFBI, transforming growth factor-beta-induced.

Fig 2 shows the workflow for the discovery, verification, and validation of plasma biomarkers that could differentiate HCA cases from non-HCA controls. Label-free LC-MS/MS analyses of 18 individual plasma samples accurately identified and quantified 256 plasma proteins (at least one unique peptide; at a 1% false discovery rate; S2 Table). Forty-two proteins (16.4%, 42/256) were identified with one unique peptide; all others were identified with two or more peptides. Among these 256 proteins, 18 proteins showed significantly different expressions between the HCA and non-HCA groups, of which 13 (72.2%) were upregulated and 5 (27.8%) were downregulated in HCA cases (S2 Fig). Hierarchical clustering analysis of these 18 DEPs revealed that their expression patterns were in general different between the non-HCA control and HCA case groups (S3 Fig; S3 Table).

### Gene ontology (GO) enrichment analysis of the identified DEPs

To gain insight into the function of the identified 18 DEPs, GO enrichment analysis was performed using DAVID (https://david.ncifcrf.gov). To gain insight into the function of the identified 18 DEPs, GO enrichment analysis was performed using DAVID (https://david.ncifcrf.gov) [40]. The top five enriched biological processes of the DEPs were protein activation cascade, phagocytosis, complement activation (classical pathway), humoral immune response, and complement activation (S4 Fig). Moreover, these pathway-related proteins were upregulated in HCA cases.

### Targeted protein verification via LC-MRM-MS analysis (verification phase)

To further assess the DEPs identified, MRM-MS analysis was performed using 40 individual plasma samples from cohort 1 (20 HCA cases and 20 non-HCA controls). S5 Fig shows the representative response curves of the heavy peptides spiked into the plasma matrix at different concentrations using the optimized MRM transitions. The relative quantification of 49 surrogate peptides from 12 of the 18 identified DEPs (except immunoglobulin-domain proteins and accidently missed TGFBI) was performed using the established LC-MRM-MS method (S4 and S5 Tables). The measured abundance of each peptide, expressed as the light (i.e., endogenous) peptide to heavy peptide PAR, was compared between the HCA and non-HCA groups. Overall, 11 peptides from three proteins (C4A, CRP, and SAA4) exhibited statistically significant changes (*P* < 0.05). Specifically, the levels of AFVFPK, ESDTSYVSLK, GYSIFSYATK, and QDNEILIFWSK from CRP; EALQGVGDMGR, AYWDIMISNHQNSNR, GNYDAAQR, GPGGVWAAK, and FRPDGLPK from SAA4; as well as VLSLAQEQVGGSPEK and QGSFQGGFR from C4A, were found to be significantly higher in the plasma of women with HCA than in the non-HCA control group (Table 1 and S6 Fig). ROC analyses of the PARs of the 11 dysregulated peptides (Table 1) showed that their AUCs ranged from 0.695 to 0.830, and did not differ significantly from each other (all variables: *P* = 0.21–0.96).

**Table 1:** Correlation between relative abundance of plasma biomarkers and histologic chorioamnionitis, with corresponding areas under the curves and optimal cutoff values for each protein.

Peptide sequence	Histologic chorioamnionitis		*P*-value	AUC	Cutoff value	Sensitivity ^a^	Specificity ^a^
	Absent (n = 20)	Present (n = 20)					
AFVFPK (CRP)	0.112 ± 0.139	0.467 ± 0.698	**0.007**	0.750	≥0.1835	55.0	90.0
ESDTSYVSLK (CRP)	0.139 ± 0.169	0.575 ± 0.876	**0.007**	0.751	≥0.2865	50.0	95.0
GYSIFSYATK (CRP)	0.062 ± 0.075	0.274 ± 0.436	**0.007**	0.748	≥0.1227	50.0	95.0
QDNEILIFWSK (CRP)	0.133 ± 0.154	0.311 ± 0.383	**0.033**	0.700	≥0.1045	68.4	70.0
EALQGVGDMGR (SAA4)	0.359 ± 0.135	0.582 ± 0.231	**0.002**	0.780	≥0.5665	65.0	95.0
AYWDIMISNHQNSNR (SAA4)	0.133 ± 0.052	0.213 ± 0.089	**0.006**	0.759	≥0.2123	68.4	94.7
GNYDAAQR (SAA4)	1.171 ± 0.369	1.857 ± 0.597	**<0.001**	0.830	≥1.5443	80.0	85.0
GPGGVWAAK (SAA4)	0.973 ± 0.339	1.488 ± 0.528	**0.001**	0.800	≥1.2296	80.0	75.0
FRPDGLPK (SAA4)	0.966 ± 0.312	1.534 ± 0.503	**<0.001**	0.828	≥1.2268	80.0	80.0
VLSLAQEQVGGSPEK (C4A)	1.063 ± 0.378	1.334 ± 0.449	**0.035**	0.695	≥0.9058	85.0	55.0
QGSFQGGFR (C4A)	0.520 ± 0.202	0.680 ± 0.222	**0.035**	0.695	≥0.4872	80.0	55.0

AUC, areas under the curves; CRP, C-reactive protein; SAA4; serum amyloid A4; C4A, complement C4-A.

Data are given as the means ± standard deviation (peak area ratio).

^a^ Values are given as %.

Unlike the results obtained from the discovery cohort, univariate analysis of the MRM-verification cohort data showed that gestational age at sampling was significantly lower in women with HCA (*P* = 0.043; Table 2), despite of the attempted matching for gestational age at sampling between the groups. Therefore, the data were adjusted for baseline differences in gestational age at sampling using multivariate logistic analyses. All continuous factors were entered as dichotomous factors in the multivariate logistic regression model using cutoff points obtained from the ROC curve analysis. The optimal cutoff values for gestational age at sampling and the PARs of AFVFPK, ESDTSYVSLK, GYSIFSYATK, QDNEILIFWSK, EALQGVGDMGR, AYWDIMISNHQNSNR, GNYDAAQR, GPGGVWAAK, FRPDGLPK, VLSLAQEQVGGSPEK, and QGSFQGGFR were ≤ 33.0 weeks, ≥ 0.1835, ≥ 0.2865, ≥ 0.1227, ≥ 0.1045, ≥ 0.5665, ≥ 0.2123, ≥ 1.5443, ≥ 1.2296, ≥ 1.2268, ≥ 0.9058, and ≥ 0.4872, respectively (Table 1). High PARs for AFVFPK, ESDTSYVSLK, GYSIFSYATK, QDNEILIFWSK, EALQGVGDMGR, AYWDIMISNHQNSNR, GNYDAAQR, GPGGVWAAK, FRPDGLPK, VLSLAQEQVGGSPEK, and QGSFQGGFR were significantly associated with the occurrence of HCA, after adjusting for low gestational age at sampling (≤ 33.0 weeks, Table 3).

**Table 2:** Demographic and clinical characteristics of women with preterm premature rupture of membranes included in the MRM verification cohort.

	Histologic chorioamnionitis		*P-*value
	Absent (n = 20)	Present (n = 20)	
Age (years)	29.7 ± 3.7	30.7 ± 4.2	0.512
Nulliparity	50.0% (10/20)	45.0% (9/20)	1.000
Gestational age at sampling (weeks)	33.0 ± 1.4	31.8 ± 1.9	**0.043**
Gestational age at delivery (weeks)	33.2 ± 1.4	32.2 ± 1.9	0.157
Male gender	45% (9/20)	50% (10/20)	0.752
Use of antibiotics	100% (20/20)	95.0% (19/20)	1.000
Use of tocolytics	35.0% (7/20)	70.0% (14/20)	0.056
Use of corticosteroids	75.0% (15/20)	75.0% (15/20)	1.000
Serum CRP (mg/dL)	0.50 ± 0.40	1.59 ± 1.91	0.041
Funisitis	0% (0/20)	35.0% (7/20)	**0.004**

MRM, multiple reaction monitoring; CRP, C-reactive protein.

Values are given as the mean ± SD or % (n).

**Table 3:** Multivariate logistic regression analysis assessing the association between the various maternal plasma proteins and histologic chorioamnionitis in women with preterm premature rupture of membranes included in the MRM verification cohort.

Variables^a^	Adjusted odds ratio (95% confidence interval)^b^	*P*-value^c^
AFVFPK (CRP)	10.410 (1.756–61.721)	**0.010**
ESDTSYVSLK (CRP)	18.072 (1.885–173.246)	**0.012**
GYSIFSYATK (CRP)	18.072 (1.885–173.246)	**0.012**
QDNEILIFWSK (CRP)	5.065 (1.197–21.439)	**0.028**
EALQGVGDMGR (SAA4)	28.434 (3.034–266.449)	**0.003**
AYWDIMISNHQNSNR (SAA4)	38.379 (3.439–428.295)	**0.003**
GNYDAAQR (SAA4)	18.342 (3.371–99.812)	**0.001**
GPGGVWAAK (SAA4)	11.734 (2.410–57.121)	**0.002**
FRPDGLPK (SAA4)	12.790 (2.481–65.945)	**0.002**
VLSLAQEQVGGSPEK (C4A)	12.109 (1.902–77.113)	**0.008**
QGSFQGGFR (C4A)	6.362 (1.300–31.138)	**0.022**

CRP, C-reactive protein; SAA4; serum amyloid A4; C4A, complement C4-A.

^a^ All continuous predictors were entered as dichotomous variables using the cut-off values derived from the receiver-operating characteristic curves to predict histologic chorioamnionitis.

^a^ Variables were dichotomized: high AFVFPK (≥ 0.1835 vs. < 0.1835), high ESDTSYVSLK (≥ 0.2865 vs. < 0.2865), high GYSIFSYATK (≥ 0.1227 vs. < 0.1227), high QDNEILIFWSK (≥ 0.1045 vs. < 0.1045), high EALQGVGDMGR (≥ 0.5665 vs. < 0.5665), high AYWDIMISNHQNSNR (≥ 0.2123 vs. < 0.2123), high GNYDAAQR (≥ 1.5443 vs. < 1.5443), high GPGGVWAAK (≥ 1.2296 vs. < 1.2296), high FRPDGLPK (≥ 1.2268 vs. < 1.2268), high VLSLAQEQVGGSPEK (≥ 0.9058 vs. < 0.9058), and high QGSFQGGFR (≥ 0.4872 vs. < 0.4872).

^b^ Adjusted for low gestational age at sampling (≤33.0 weeks).

^c^ For the adjusted odds ratio.

### Multiple-marker panel as predictor of HCA

**Fig 3:**
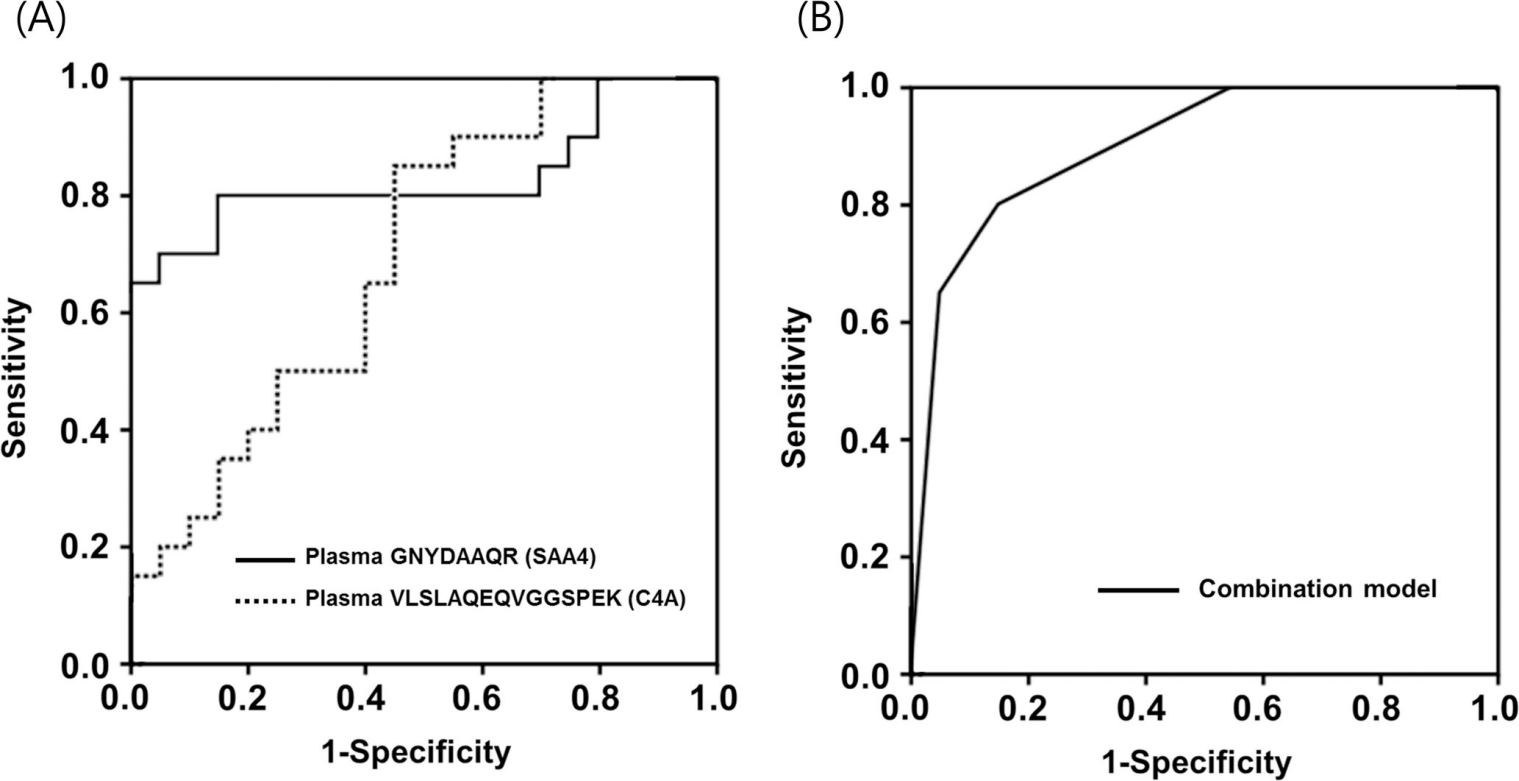
**(A)** ROC curves for three plasma GNYDAAQR (SAA4) and VLSLAQEQVGGSPEK (C4A) levels to predict HCA (area under the curve [AUC] ± standard error [SE] = 0.830 ± 0.071 and 0.695 ± 0.084, respectively). **(B)** ROC curve for the best combined predictive model [including plasma VLSLAQEQVGGSPEK (C4A) and GNYDAAQR (SAA4)] to predict HCA, with an AUC = 0.899 (*P* < 0.05 for VLSLAQEQVGGSPEK vs. the combined predictive model, and *P* = 0.19 for GNYDAAQR vs. the combined predictive model). C4A, complement C4-A; HCA, histologic chorioamnionitis; ROC, receiver operating characteristic; SAA4; serum amyloid A4.

To develop the best multiple-marker panel for the MRM-MS dataset, based on the combination of the identified biomarker candidates, multivariate analysis with a forward selection was performed on three biomarker candidates with *P* < 0.05 from the univariate analysis. As described in the statistical analysis section, only one peptide per protein, which was selected based on the highest value of AUCs of different peptides, was included in this stepwise regression model. Peptides ESDTSYVSLK, GNYDAAQR, and VLSLAQEQVGGSPEK were selected for CRP, SAA4, and C4A proteins, respectively, and included in the stepwise regression analysis. A two-marker panel for the diagnosis of HCA, consisting of high PARs for GNYDAAQR (SAA4 ≥ 1.5443) and VLSLAQEQVGGSPEK (C4A ≥ 0.9058), was identified as the best combination of multiple markers (Table 4), with AUC values of 0.899 (95% confidence interval [CI]: 0.802–0.995; *P* = 0.796 by Hosmer-Lemeshow test). Moreover, a cutoff value of ≥ 0.43 was identified as the optimal threshold, with a sensitivity of 80% and a specificity of 85% for the diagnosis of HCA. The AUC for this multiple-marker panel was significantly larger than the AUC for VLSLAQEQVGGSPEK (C4A), but not GNYDAAQR (SAA4) (*P* = 0.012 and *P* = 0.196, respectively; Fig 3).

**Table 4:** Regression coefficients, odds ratios, and 95% confidence intervals of the best protein panel* for predicting histologic chorioamnionitis.

Predictor	Beta-coefficient	SE	OR (95% CI)	*P*-value
High GNYDAAQR (SAA4) (≥ 1.5443)^†^	3.686	1.146	39.899 (4.219–377.330)	0.001
High VLSLAQEQVGGSPEK (C4A) (≥ 0.9058)^†^	2.703	1.213	14.929 (1.385–160.953)	0.026
Constant	-3.497	1.253	0.030	0.005

SE, standard error; OR, odds ratio; CI, confidence interval; SAA4; serum amyloid A4; C4A, complement C4-A.

^†^Variables were dichotomized: high GNYDAAQR (≥ 1.5443 vs. < 1.5443) and high VLSLAQEQVGGSPEK (≥ 0.9058 vs. < 0.9058).

*Formula that was generated to predict histologic chorioamnionitis was as follows: Y = logₑ (Z) = -3.497 + 3.686 (if GNYDAAQR was ≥ 1.5443) + 2.703 (if VLSLAQEQVGGSPEK was ≥ 0.9058). Z = eʸ and risk (%) = [Z/(1 + Z)]× 100.

### Validation of protein biomarkers by ELISA

**Fig 4:**
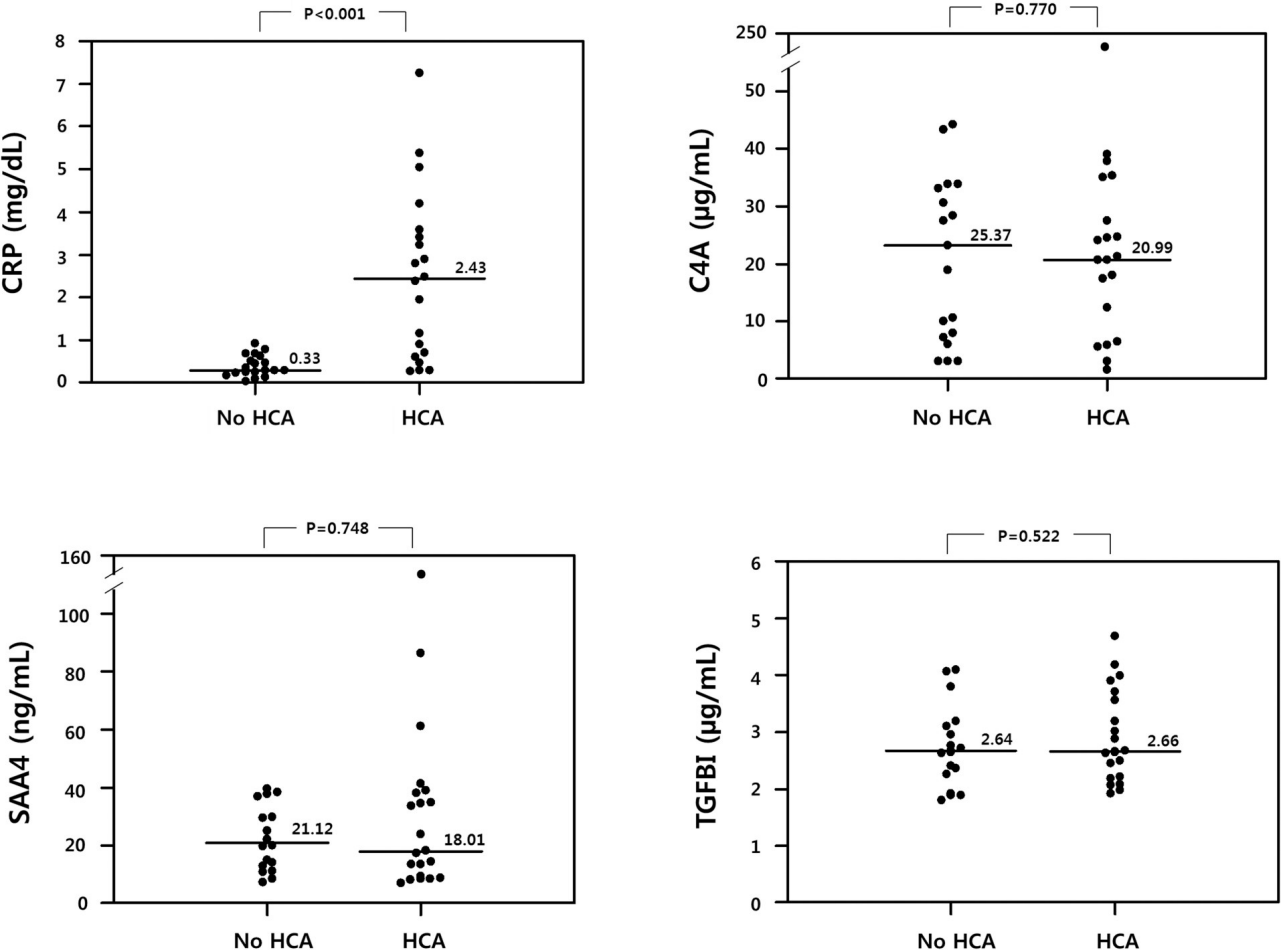
Plasma levels of C-reactive protein (CRP), complement C4-A (C4A), serum amyloid A4 (SAA4), and transforming growth factor-beta-induced (TGFBI) in histologic chorioamnionitis (HCA) cases and non-HCA controls. All samples analysed (n = 20 and 18, respectively) were from study cohort 2. Horizontal lines indicate the respective median values.

A validation study was also performed in an independent study group (n = 38, cohort 2) for C4A, SAA4, and CRP, which were significantly highly expressed in HCA cases compared with non-HCA controls in the targeted proteomic analysis. As observed in the targeted proteomics, the median plasma levels of CRP were significantly higher in women with HCA than in those without HCA (*P* < 0.001, Fig 4), and this difference remained significant even after adjusting for gestational age at sampling (odds ratio [OR]: 7.53; 95% CI: 1.10–51.34; *P* = 0.039) (S6 Table). However, the plasma levels of C4A and SAA4, as determined by ELISA, did not significantly differ between the HCA and non-HCA groups in cohort 2 and the entire study cohort (Fig 4 and S7 Table). For TGFBI, which was inadvertently missed in the targeted proteomic assay, its plasma levels determined by ELISA did not significantly differ between the non-HCA and HCA groups within cohorts 1 and 2 (Fig 4), and the total study cohort (S7 Table).

## Discussion

This study aimed to identify the potential biomarkers in blood plasma that are associated with HCA in patients with PPROM and to develop a multimarker panel to improve discriminatory power by combining these biomarker candidates. Overall, this study identified 18 DEPs using label-free LC-MS/MS analysis in peripheral plasma samples of women with PPROM and HCA and characterized their potential biological pathways. Moreover, among 12 DEPs verified by MRM-MS analysis, three plasma proteins (CRP, C4A, and SAA4) were confirmed as potential independent biomarkers for the detection of HCA, which allowed to develop a two-marker panel (with AUC values of 0.899, comprising GNYDAAQR and VLSLAQEQVGGSPEK peptides of SAA4 and C4A, respectively) that could help improve the prediction of HCA. Lastly, CRP, C4A, and SAA4 were further assessed in plasma samples from an independent cohort of women with PPROM using ELISA, but only CRP was detected at significantly higher concentrations in PPROM women with HCA compared with those without HCA. Using MS-based proteomic technology, this study provides new insights for a better understanding of the molecular responses and biochemical mechanisms underlying acute inflammatory processes in the placenta that could be detected in the maternal circulation.

In the context of PPROM, an ideal HCA prediction tool would accurately and noninvasively identify women at higher risk. In the present study, the discriminatory ability of plasma C4A, SAA4, and CRP ranged between AUC 0.695–0.830 as single markers for noninvasively classifying HCA, suggesting that any single marker alone is not sufficiently useful in clinical practice. The best protein multi-marker panel herein identified resulted from the combination of C4A and SAA4, but only marginally improved HCA risk prediction compared with plasma SAA4 alone, as shown by a small change in AUC values (0.899 vs. 0.830, *P* = 0.196). Baseline demographic and clinical factors, such as gestational age at sampling, parity, and amniotic fluid index, were also strongly associated with the occurrence of HCA [14, 41]; thus, it could be useful to assess whether the herein identified proteomic plasma biomarkers in combination with these clinical factors could significantly improve the prediction of HCA.

Using proteomic shotgun and targeted approaches, SAA4, C4A, and CRP were identified as potential noninvasive biomarkers for the detection of HCA in patients with PPROM. C4A is a protein that belongs to the anaphylatoxin family (C3A, C4A, and C5A) produced by the activation of the complement system, which plays an important role in immune protection, immune tolerance, and immune complex clearance. C4A is known to be expressed in the placenta [42, 43]. Consistent with the known function and expression site of C4A, in the context of preterm labor, previous cross-sectional studies revealed that elevated levels of C4A in AF (but not in plasma) were significantly increased in patients with MIAC compared to those without MIAC [44, 45]. However, to date, no studies have investigated whether altered C4A levels in the plasma are associated with the development of HCA in PPROM. The present study provides evidence for a causal association between elevated plasma C4A levels and HCA using a targeted proteomic approach, but this association should be further confirmed by additional studies with larger patient cohorts.

SAA4, also known as a constitutive SAA (or C-SAA), is a novel member of the SAA superfamily that is synthesized constitutively in the liver, and is a minor acute-phase reactant in humans [46, 47]. It is also expressed in the placenta, including the decidua and villous tissue [48]. In contrast to SAA4, SAA1 and SAA2 (A-SAA) respond to acute phase reactions as a major acute-phase reactant during inflammation [49]. Data from a previous study comprising a small sample size (n = 43) showed that serum SAA levels, as determined by ELISA, were significantly elevated in women with PPROM and HCA than in those without HCA [50]. Nevertheless, in the available reports, the expression of the various SAA subtypes, especially SAA4, has not yet been investigated in women with PPROM concerning subclinical HCA and MIAC. This study demonstrated for the first time, using MRM-MS analyses, that SAA4 levels were significantly increased in the plasma of women with PPROM and HCA. These findings are, in part, supported by previous studies demonstrating that SAA4 may play a role in inflammation-related atherogenesis and that changes in serum SAA4 levels were associated with inflammatory activities in patients undergoing renal allograft transplantation, although the magnitude of changes was not larger than a 3-fold increase [47, 51].

CRP is a protein that is expressed during inflammation or tissue injury (the acute-phase response) with a peak within 2–3 days of illness, and is produced primarily by the liver in response to inflammatory mediators, particularly interleukin-6 [52]. In the obstetric field, several studies have explored the role of serum CRP as a predictor of HCA and/or MIAC. Elevated CRP levels in circulation were found to be associated with these two outcomes in women with PPROM, although these associations were weak and conflicting [7, 14, 53, 54]. In particular, a recent study with a large cohort of PPROM patients (n = 386) showed a weak association between the occurrence of HCA and elevated serum CRP levels, which is in agreement with the findings of the present study [54]. The discrepant result for CRP among studies is most likely related to the eligibility criteria of each study cohort, in particular, regarding time interval between maternal blood and placental samplings. In particular, Ray et al. reported that plasma levels of CRP progressively and significantly increased in HCA patients, with a difference from the non‐HCA group becoming statistically significant 2 days before delivery [41].

The present study identifies an important question regarding elucidating mechanisms underlying the alteration of the expressions of C4A, SAA4, and CRP in maternal plasma mediated by acute HCA. We speculate that the mechanisms are likely to be associated with the location where C4A, SAA4, and CRP are produced. Typically, local infection/inflammation (i.e., MIAC/intra-amniotic inflammation in utero, as well as “sterile” intra-amniotic inflammation) has been considered to be the cause of acute HCA [16]. Thus, the levels of proteins that are mainly produced in the liver in response to the infectious/inflammatory signal in the amniotic cavity can be elevated in the maternal circulation. In contrast, the levels of proteins that are produced only by immune cells at the original site of infection (i.e., amniotic cavity) can be increased in the local areas (e.g., AF and fetal membranes) surrounding to the site at which they are originally produced but not in the maternal plasma. In fact, the liver is the main organ that can synthesize and secrete C4A, SAA4, and CRP in response to local inflammatory stimuli [most prominently IL-6 for acute-phase proteins (e.g., CRP and SAA4)] [46, 47, 52, 55].

In the current study, only one out of the three proteins identified by targeted proteomics was validated by ELISA, which could be due to (1) the relatively small sample size; (2) a greater difference of gestational age at sampling between the case and control groups in the cohort 2 compared with that in cohort 1. Additionally, compared with MRM-MS proteomic assays, ELISA has fundamental flaws, including a comparatively lower sensitivity to measure protein concentrations, cross-reactivity of the antibodies with non-target antigens, high-dose hook effect, and the likelihood of interference of autoantibodies and anti-reagent antibodies [56]. Despite several advantages of MRM assays over ELISAs, the following several issues could affect the accuracy of MRM-based quantitation: (1) inappropriate fraction preparation of the complex mixture containing other proteins with high abundance (i.e., serum or plasma), which may lead to the presence of interfering molecules in the mixture [57]; incomplete digestion of proteins with trypsin [58]; (3) and the presence of artifactual peptide modifications (i.e., carbamylation, oxidation) [57]. Consequently, these two assays (MRM and ELISA) are complementary to each other; however, they may not replace one another [59]. This discrepancy could also be attributed to the fact that the MRM quantitation was based on individual peptides, whereas the ELISA quantification was performed using antibodies targeting specific epitopes on the surface of whole protein molecules.

In the present study, GO enrichment analysis revealed that the top-ranked biological processes detected in the maternal plasma related to the development of HCA were protein activation cascade, phagocytosis, complement activation (classical pathway), and humoral immune response. Previous pre-clinical *in vivo* studies have implicated the protein activation cascade in the pathogenesis of intestinal inflammation and bacterial infection [60, 61]. In addition, a recent study using AF samples has shown that regulation of the protein activation cascade is significantly involved in the pathogenesis of late PPROM complicated by both HCA and MIAC [62]. A recent study using *ex vivo* phagocytosis assays, and scanning and transmission electron microscopy showed that phagocytosis by AF neutrophils was observed in the amniotic cavity of patients with intra-amniotic infection, suggesting that neutrophil phagocytosis is an important host defense mechanism for killing microbes invading the amniotic cavity [63]. It is well acknowledged that the complement activation cascade is highly associated to enhanced inflammation and immunity [64]. Collectively, the present data on biological functional pathways suggested that infection, inflammation, and immune cascades that took place simultaneously in the maternal circulation were most importantly associated with PPROM complicated by HCA. Finally, proteins identified previously in a recent proteomic study conducted by Pan et al. were not found in the present study [65]. However, this discrepancy is quite typical because these two studies were different in terms of outcomes of interests (preterm birth vs. HCA), disease entity studied (mixed population of preterm, term, and PPROM women vs. PPROM women), type of samples (fetal membranes and placental villi vs. plasma), study design, and criteria for inclusion and exclusion.

Limitations of the current study include its retrospective study design conducted at a single center, and relatively small sample size. Moreover, the plasma samples were gathered at a single time point; thus, it was not possible to identify a specific pre-delivery time point when single measurement of biomarkers in plasma could be recommended for the best prediction of HCA. Thus, the interpretation of our findings should be treated with caution because our study included only women who delivered within 96 h after collection of plasma samples, hence providing information on the biomarkers associated with the development of HCA up to 4 days before delivery but not during the remaining pregnancy period after PPROM. Another limitation of the study was that plasma concentrations of immunoglobulin were not measured for the verification study; thus, the present study could not provide any information on immunoglobulin-related significant molecules associated with HCA in women with PPROM. Furthermore, during the ELISA validation experiments, the association of two (66%) of the three proteins identified by MRM-MS was not reproduced in the ELISA results, as in other previous studies [59, 66]. Of course, different quantitation results have been reported in the MRM studies conducted using selected peptides derived from the same target proteins [24, 58, 67], and different results of proteins quantitation based on the different immunoassay techniques have been also reported [68, 69]. Other limitations of this study included the use of (1) 2 μL of each plasma sample for protein digestion during sample preparation for discovery proteomics, which may affect final protein quantification, and (2) crude plasma samples without depletion of high-abundance proteins, leading to a smaller number of proteins identified in the discovery phase of our study. However, these issues may not affect the interpretation of results in this study, according to the recent report published by Geyer et al. The said study showed a newly introduced proteomic technology with no protein depletion from crude plasma sample volume, thereby leading to the development of a rapid and robust ‘‘plasma proteome profiling” pipeline [70]. Moreover, several studies have demonstrated that depletion strategies to remove high-abundance proteins in plasma could lead to the concomitant removal of some non-targeted proteins (e.g., proteins bound to albumin and immunoglobulins) that may be of potential interest as candidate biomarkers, which may introduce bias in proteomic studies [71, 72].

Nevertheless, to the best of our knowledge, this is the first comprehensive proteomic study of maternal plasma to identify potential biomarkers for HCA in women with PPROM. Herein, both ELISA and MRM-MS were used to verify candidate target proteins in plasma, providing an opportunity to compare the results of these two techniques. In addition, label-free quantification for biomarker discovery, particularly by analyzing individual samples, allowed to identify quantitative differences between low abundance proteins, as well as for detecting small changes between samples from an individual [20]. Finally, we included only women who delivered within 96 h after collection of plasma samples, allowing for direct comparison of plasma biomarkers and placental histology. Latency of 96 hours was adopted as a criterion for the study because the median maternal blood levels of various inflammatory markers (i.e., IL-6, G-CSF, CRP, and WBC) were significantly higher in the HCA group than in the non-HCA group for up to the 3 days preceding delivery in women with PPROM [41, 73, 74] and the number of PPROM women who delivered within 72 hours after plasma sampling was too small in our dataset to warrant analysis. The longer the latency period (time between plasma sampling and delivery) in cases of PPROM, the stronger is the effect on the placental pathology with respect to inflammation attributed to ascending infection via ruptured amniotic membranes. Several studies investigating women with PPROM have reported that antibiotic therapy (especially clarithromycin) may attenuate or even resolve acute HCA, MIAC, and intra-amniotic inflammation in a subset of cases [75–77]. Thus, non-invasive biomarkers identified in the present study may be of clinical importance for the decision-making process with respect to the management of PPROM (re-initiation of antibiotics and expediting delivery), as they can aid in the serial evaluation of the development of acute HCA in women with PPROM.

## Conclusions

In summary, plasma C4A, SAA4, and CRP were identified as potential independent biomarkers for the detection of HCA in women with PPROM, based on targeted and shotgun proteomic analyses, showing good accuracy when used as a combined dual-biomarker panel (C4A and SAA4). Although, these proteins may have substantial biological interest as useful biomarkers for HCA, ELISA validation of these proteins, except for CRP, may not yield clinically useful predictive data. Further studies are needed to prospectively validate the clinical utility of plasma C4a and SAA4 as HCA markers in larger and different cohorts of women with PPROM. Moreover similar proteomic analyses using other noninvasive samples (such as cervicovaginal fluid and saliva) are warranted to identify HCA markers in PPROM. Collectively, the development of these non-invasive tests (including plasma biomarkers based on MRM-MS in the present study) would be very helpful, as it would allow the clinicians to target a particular subgroup of women at a high risk of acute HCA for therapeutic intervention (e.g., clarithromycin and expediting delivery) [76, 77] and in clinical trials, as well as to monitor whether the development of new HCA can be attributed to ascending infection via ruptured membranes.

## Supporting information

S1 Table Demographic and clinical characteristics of women with preterm premature rupture of membrane involved in the discovery cohort study. (XLSX)

S2 Table List of identified proteins from control (non-HCA, n = 9) and case (HCA, n = 9) groups. (XLSX)

S3 Table List of plasma proteins that exhibited statistically significant differences in a pairwise comparison of HCA vs. non-HCA in women with PPROM, using label-free quantitative analyses. (XLSX)

S4 Table List of surrogate peptides for MRM method development. (XLSX)

S5 Table Optimized MRM method parameters of 334 transitions for 49 peptides from 12 proteins. (XLSX)

S6 Table Demographic and clinical characteristics of women with preterm premature rupture of membranes included in the ELISA validation cohort. (XLSX)

S7 Table Demographic and clinical characteristics of women with preterm premature rupture of membranes included in the total cohort (n = 78). (XLSX)

S1 Fig Principal component analysis (PCA) plot. (TIF)

S2 Fig Volcano plot constructed from label-free quantification data. Volcano plot shows the plasma upregulated and downregulated differentially expressed proteins (DEPs) in the histologic chorioamnionitis (HCA) and non-HCA groups. Representative protein identifiers in red indicate statistically significant DEPs (*P* < 0.05).
(TIF)

S3 Fig Heatmap with dendrogram of hierarchical clustering analysis of 18 statistically significant differentially expressed proteins (DEPs) between histologic chorioamnionitis (HCA) case and non-HCA control groups (red = increased, green = decreased). (TIF)

S4 Fig Top five gene ontology (GO) biological process terms obtained from DAVID GO enrichment analysis for the 18 differentially expressed proteins. (TIF)

S5 Fig Representative response curves of the heavy peptides. The peptides correspond to FRPDGLPK and QGSFQGGFR from SAA4 and C4A, respectively. For each concentration point, triplicates were analyzed. C4A, complement C4-A; SAA4, serum amyloid A4.
(TIF)

S6 Fig Interactive plots for significantly upregulated peptides (from CRP, C4A, and SAA4) in plasma from women with PPROM in HCA group (case), as verified by MRM analysis. Interactive plots were generated using the normalized peak area of each MRM target peptide. The median levels of CRP, C4A, and SAA4 in plasma were significantly higher in women with HCA than in the non-HCA control group. The horizontal line in each figure represents the median value. C4A, complement C4-A; CRP, C-reactive protein; HCA, histologic chorioamnionitis; PPROM, preterm premature rupture of membranes; SAA4, serum amyloid A4.
(TIF)

S1 File Raw data for the exploratory cohort. (SAV)

S2 File Raw data for the MRM verification cohort. (SAV)

S3 File Raw data for the ELISA validation cohort. (SAV)

S4 File Raw data for the total cohort. (SAV)

S1 Text Materials and methods (the methods for placental tissue collection and processing for histologic evaluation; management of PPROM; plasma sample preparation; LC-MS/MS analysis (discovery phase); data processing for label-free quantification; statistical analysis (Discovery); plasma sample preparation for quantification of target peptides with LC-MRM-MS; LC-MRM-MS analysis; optimization of the multiplexed MRM-MS method for the quantification of target proteins using heavy isotope-labeled peptides; analysis of complement C4A and SAA4 in the plasma samples during validation phase; for further details on the inclusion of study participants employed for proteomic studies in the discovery and verification stages). (DOCX)

## References

[1] Goldenberg RL, Culhane JF, Iams JD, Romero R. Epidemiology and causes of preterm birth. Lancet. 2008;371(9606):75–84. doi: 10.1016/S0140-6736(08)60074-4 . 18177778 PMC7134569

[2] Menon R, Richardson LS. Preterm prelabor rupture of the membranes: A disease of the fetal membranes. Semin Perinatol. 2017;41(7):409–19. doi: 10.1053/j.semperi.2017.07.012 . 28807394 PMC5659934

[3] Etyang AK, Omuse G, Mukaindo AM, Temmerman M. Maternal inflammatory markers for chorioamnionitis in preterm prelabour rupture of membranes: a systematic review and meta-analysis of diagnostic test accuracy studies. Syst Rev. 2020;9(1):141. doi: 10.1186/s13643-020-01389-4 . 32532314 PMC7293113

[4] Kacerovsky M, Pliskova L, Bolehovska R, Musilova I, Hornychova H, Tambor V, et al. The microbial load with genital mycoplasmas correlates with the degree of histologic chorioamnionitis in preterm PROM. Am J Obstet Gynecol. 2011;205(3):213 e1-7. doi: 10.1016/j.ajog.2011.04.028 . 21663889

[5] Wu YW, Colford JM Jr., Chorioamnionitis as a risk factor for cerebral palsy: A meta-analysis. Jama. 2000;284(11):1417–24. doi: 10.1001/jama.284.11.1417 . 10989405

[6] Yoon BH, Romero R, Kim CJ, Jun JK, Gomez R, Choi JH, et al. Amniotic fluid interleukin-6: a sensitive test for antenatal diagnosis of acute inflammatory lesions of preterm placenta and prediction of perinatal morbidity. Am J Obstet Gynecol. 1995;172(3):960–70. doi: 10.1016/0002-9378(95)90028-4 7892891

[7] Oh KJ, Park KH, Kim SN, Jeong EH, Lee SY, Yoon HY. Predictive value of intra-amniotic and serum markers for inflammatory lesions of preterm placenta. Placenta. 2011;32(10):732–6. doi: 10.1016/j.placenta.2011.07.080 . 21839511

[8] Kim SA, Park KH, Lee SM. Non-Invasive Prediction of Histologic Chorioamnionitis in Women with Preterm Premature Rupture of Membranes. Yonsei Med J. 2016;57(2):461–8. doi: 10.3349/ymj.2016.57.2.461 . 26847301 PMC4740541

[9] Miyazaki K, Furuhashi M, Ishikawa K, Tamakoshi K, Hayashi K, Kai A, et al. Impact of chorioamnionitis on short- and long-term outcomes in very low birth weight preterm infants: the Neonatal Research Network Japan. J Matern Fetal Neonatal Med. 2016;29(2):331–7. doi: 10.3109/14767058.2014.1000852 . 25567563

[10] Park JW, Park KH, Jung EY. Clinical significance of histologic chorioamnionitis with a negative amniotic fluid culture in patients with preterm labor and premature membrane rupture. PloS one. 2017;12(3):e0173312. doi: 10.1371/journal.pone.0173312 . 28278303 PMC5344397

[11] Cobo T, Kacerovsky M, Palacio M, Hornychova H, Hougaard DM, Skogstrand K, et al. A prediction model of histological chorioamnionitis and funisitis in preterm prelabor rupture of membranes: analyses of multiple proteins in the amniotic fluid. J Matern Fetal Neonatal Med. 2012;25(10):1995–2001. doi: 10.3109/14767058.2012.666592 . 22372866

[12] Dulay AT, Buhimschi IA, Zhao G, Bahtiyar MO, Thung SF, Cackovic M, et al. Compartmentalization of acute phase reactants Interleukin-6, C-Reactive Protein and Procalcitonin as biomarkers of intra-amniotic infection and chorioamnionitis. Cytokine. 2015;76(2):236–43. doi: 10.1016/j.cyto.2015.04.014 . 25957466 PMC4824401

[13] Lee SM, Park KH, Jung EY, Kook SY, Park H, Jeon SJ. Inflammatory proteins in maternal plasma, cervicovaginal and amniotic fluids as predictors of intra-amniotic infection in preterm premature rupture of membranes. PloS one. 2018;13(7):e0200311. doi: 10.1371/journal.pone.0200311 . 29979758 PMC6034889

[14] Park JW, Park KH, Lee JE, Kim YM, Lee SJ, Cheon DH. Antibody Microarray Analysis of Plasma Proteins for the Prediction of Histologic Chorioamnionitis in Women With Preterm Premature Rupture of Membranes. Reprod Sci. 2019;26(11):1476–84. doi: 10.1177/1933719119828043 . 30727818

[15] McNamara MF, Wallis T, Qureshi F, Jacques SM, Gonik B. Determining the maternal and fetal cellular immunologic contributions in preterm deliveries with clinical or subclinical chorioamnionitis. Infect Dis Obstet Gynecol. 1997;5(4):273–9. doi: 10.1155/S1064744997000471 . 18476151 PMC2364556

[16] Kim CJ, Romero R, Chaemsaithong P, Chaiyasit N, Yoon BH, Kim YM. Acute chorioamnionitis and funisitis: definition, pathologic features, and clinical significance. Am J Obstet Gynecol. 2015;213(4 Suppl):S29–52. doi: 10.1016/j.ajog.2015.08.040 . 26428501 PMC4774647

[17] Lee SD, Kim MR, Hwang PG, Shim SS, Yoon BH, Kim CJ. Chorionic plate vessels as an origin of amniotic fluid neutrophils. Pathol Int. 2004;54(7):516–22. doi: 10.1111/j.1440-1827.2004.01659.x . 15189506

[18] Dawe GS, Tan XW, Xiao ZC. Cell migration from baby to mother. Cell Adh Migr. 2007;1(1):19–27. . 19262088 PMC2633676

[19] Sheller-Miller S, Choi K, Choi C, Menon R. Cyclic-recombinase-reporter mouse model to determine exosome communication and function during pregnancy. Am J Obstet Gynecol. 2019;221(5):502 e1– e12. doi: 10.1016/j.ajog.2019.06.010 . 31207235

[20] Dowle AA, Wilson J, Thomas JR. Comparing the Diagnostic Classification Accuracy of iTRAQ, Peak-Area, Spectral-Counting, and emPAI Methods for Relative Quantification in Expression Proteomics. J Proteome Res. 2016;15(10):3550–62. doi: 10.1021/acs.jproteome.6b00308 . 27546623

[21] Pereira L, Reddy AP, Alexander AL, Lu X, Lapidus JA, Gravett MG, et al. Insights into the multifactorial nature of preterm birth: proteomic profiling of the maternal serum glycoproteome and maternal serum peptidome among women in preterm labor. Am J Obstet Gynecol. 2010;202(6):555 e1–10. doi: 10.1016/j.ajog.2010.02.048 . 20413102

[22] Esplin MS, Merrell K, Goldenberg R, Lai Y, Iams JD, Mercer B, et al. Proteomic identification of serum peptides predicting subsequent spontaneous preterm birth. Am J Obstet Gynecol. 2011;204(5):391 e1–8. doi: 10.1016/j.ajog.2010.09.021 . 21074133 PMC3103758

[23] Parry S, Zhang H, Biggio J, Bukowski R, Varner M, Xu Y, et al. Maternal serum serpin B7 is associated with early spontaneous preterm birth. Am J Obstet Gynecol. 2014;211(6):678 e1–12. doi: 10.1016/j.ajog.2014.06.035 . 24954659 PMC4254341

[24] Keshishian H, Addona T, Burgess M, Kuhn E, Carr SA. Quantitative, multiplexed assays for low abundance proteins in plasma by targeted mass spectrometry and stable isotope dilution. Mol Cell Proteomics. 2007;6(12):2212–29. doi: 10.1074/mcp.M700354-MCP200 . 17939991 PMC2435059

[25] Thomas CE, Sexton W, Benson K, Sutphen R, Koomen J. Urine collection and processing for protein biomarker discovery and quantification. Cancer Epidemiol Biomarkers Prev. 2010;19(4):953–9. doi: 10.1158/1055-9965.EPI-10-0069 20332277 PMC2852495

[26] Tambor V, Kacerovsky M, Lenco J, Bhat G, Menon R. Proteomics and bioinformatics analysis reveal underlying pathways of infection associated histologic chorioamnionitis in pPROM. Placenta. 2013;34(2):155–61. doi: 10.1016/j.placenta.2012.11.028 . 23246098

[27] Tambor V, Kacerovsky M, Andrys C, Musilova I, Hornychova H, Pliskova L, et al. Amniotic fluid cathelicidin in PPROM pregnancies: from proteomic discovery to assessing its potential in inflammatory complications diagnosis. PloS one. 2012;7(7):e41164. doi: 10.1371/journal.pone.0041164 . 22815956 PMC3399859

[28] Buhimschi IA, Zambrano E, Pettker CM, Bahtiyar MO, Paidas M, Rosenberg VA, et al. Using proteomic analysis of the human amniotic fluid to identify histologic chorioamnionitis. Obstet Gynecol. 2008;111(2 Pt 1):403–12. doi: 10.1097/AOG.0b013e31816102aa . 18238979

[29] Park JW, Park KH, Jung EY, Cho SH, Jang JA, Yoo HN. Short cervical lengths initially detected in mid-trimester and early in the third trimester in asymptomatic twin gestations: Association with histologic chorioamnionitis and preterm birth. PloS one. 2017;12(4):e0175455. doi: 10.1371/journal.pone.0175455 . 28399138 PMC5388475

[30] Jung EY, Choi BY, Rhee J, Park J, Cho SH, Park KH. Relation between amniotic fluid infection or cytokine levels and hearing screen failure in infants at 32 wk gestation or less. Pediatr Res. 2017;81(2):349–55. doi: 10.1038/pr.2016.219 . 27925622

[31] Park KH, Kim SN, Oh KJ, Lee SY, Jeong EH, Ryu A. Noninvasive prediction of intra-amniotic infection and/or inflammation in preterm premature rupture of membranes. Reprod Sci. 2012;19(6):658–65. doi: 10.1177/1933719111432869 . 22457430

[32] Ryu A, Park KH, Oh KJ, Lee SY, Jeong EH, Park JW. Predictive value of combined cervicovaginal cytokines and gestational age at sampling for intra-amniotic infection in preterm premature rupture of membranes. Acta Obstet Gynecol Scand. 2013;92(5):517–24. doi: 10.1111/aogs.12073 . 23324124

[33] Gibbs RS, Blanco JD, St Clair PJ, Castaneda YS. Quantitative bacteriology of amniotic fluid from women with clinical intraamniotic infection at term. J Infect Dis. 1982;145(1):1–8. doi: 10.1093/infdis/145.1.1 7033397

[34] Cox J, Mann M. MaxQuant enables high peptide identification rates, individualized p.p.b.-range mass accuracies and proteome-wide protein quantification. Nat Biotechnol. 2008;26(12):1367–72. doi: 10.1038/nbt.1511 . 19029910

[35] Tyanova S, Temu T, Cox J. The MaxQuant computational platform for mass spectrometry-based shotgun proteomics. Nat Protoc. 2016;11(12):2301–19. doi: 10.1038/nprot.2016.136 . 27809316

[36] Tyanova S, Temu T, Sinitcyn P, Carlson A, Hein MY, Geiger T, et al. The Perseus computational platform for comprehensive analysis of (prote)omics data. Nat Methods. 2016;13(9):731–40. doi: 10.1038/nmeth.3901 . 27348712

[37] Cox J, Neuhauser N, Michalski A, Scheltema RA, Olsen JV, Mann M. Andromeda: a peptide search engine integrated into the MaxQuant environment. J Proteome Res. 2011;10(4):1794–805. doi: 10.1021/pr101065j . 21254760

[38] Vandenbroucke JP, von Elm E, Altman DG, Gotzsche PC, Mulrow CD, Pocock SJ, et al. Strengthening the Reporting of Observational Studies in Epidemiology (STROBE): explanation and elaboration. PLoS Med. 2007;4(10):e297. doi: 10.1371/journal.pmed.0040297 . 17941715 PMC2020496

[39] DeLong ER, DeLong DM, Clarke-Pearson DL. Comparing the areas under two or more correlated receiver operating characteristic curves: a nonparametric approach. Biometrics. 1988;44(3):837–45. . 3203132

[40] Huang da W, Sherman BT, Lempicki RA. Systematic and integrative analysis of large gene lists using DAVID bioinformatics resources. Nat Protoc. 2009;4(1):44–57. doi: 10.1038/nprot.2008.211 . 19131956

[41] Le Ray I, Mace G, Sediki M, Lirussi F, Riethmuller D, Lentz N, et al. Changes in maternal blood inflammatory markers as a predictor of chorioamnionitis: a prospective multicenter study. Am J Reprod Immunol. 2015;73(1):79–90. doi: 10.1111/aji.12323 . 25263526

[42] Dodds AW, Ren XD, Willis AC, Law SK. The reaction mechanism of the internal thioester in the human complement component C4. Nature. 1996;379(6561):177–9. doi: 10.1038/379177a0 8538770

[43] Lokki AI, Heikkinen-Eloranta J, Jarva H, Saisto T, Lokki ML, Laivuori H, et al. Complement activation and regulation in preeclamptic placenta. Front Immunol. 2014;5:312. doi: 10.3389/fimmu.2014.00312 . 25071773 PMC4088925

[44] Soto E, Romero R, Richani K, Yoon BH, Chaiworapongsa T, Vaisbuch E, et al. Evidence for complement activation in the amniotic fluid of women with spontaneous preterm labor and intra-amniotic infection. J Matern Fetal Neonatal Med. 2009;22(11):983–92. doi: 10.3109/14767050902994747 . 19900036 PMC3437778

[45] Soto E, Romero R, Richani K, Espinoza J, Nien JK, Chaiworapongsa T, et al. Anaphylatoxins in preterm and term labor. J perinatal med. 2005;33(4):306–13. doi: 10.1515/JPM.2005.051 . 16207115 PMC1472833

[46] De Buck M, Gouwy M, Wang JM, Van Snick J, Opdenakker G, Struyf S, et al. Structure and Expression of Different Serum Amyloid A (SAA) Variants and their Concentration-Dependent Functions During Host Insults. Curr Med Chem. 2016;23(17):1725–55. doi: 10.2174/0929867323666160418114600 . 27087246 PMC5405626

[47] Yamada T, Miyake N, Itoh K, Igari J. Further characterization of serum amyloid A4 as a minor acute phase reactant and a possible nutritional marker. Clin Chem Lab Med. 2001;39(1):7–10. doi: 10.1515/CCLM.2001.003 . 11256804

[48] Sandri S, Urban Borbely A, Fernandes I, de Oliveira EM, Knebel FH, Ruano R, et al. Serum amyloid A in the placenta and its role in trophoblast invasion. PloS one. 2014;9(3):e90881. doi: 10.1371/journal.pone.0090881 . 24614130 PMC3948705

[49] Uhlar CM, Whitehead ASJEjob. Serum amyloid A, the major vertebrate acute‐phase reactant. Eur J Biochem. 1999;265(2):501–23. 10504381 10.1046/j.1432-1327.1999.00657.x

[50] Cekmez Y, Cekmez F, Özkaya E, Pirgon Ö, Yılmaz Z, Yılmaz EA, et al. Proadrenomedullin and serum amyloid A as a predictor of subclinical chorioamnionitis in preterm premature rupture of membranes. J Interferon Cytokine Res. 2013;33(11):694–9. doi: 10.1089/jir.2012.0134 24010826

[51] Meek RL, Urieli-Shoval S, Benditt EP. Expression of apolipoprotein serum amyloid A mRNA in human atherosclerotic lesions and cultured vascular cells: implications for serum amyloid A function. Proc Natl Acad Sci U S A. 1994;91(8):3186–90. doi: 10.1073/pnas.91.8.3186 8159722 PMC43540

[52] Sproston NR, Ashworth JJ. Role of C-Reactive Protein at Sites of Inflammation and Infection. Front Immunol. 2018;9:754. doi: 10.3389/fimmu.2018.00754 . 29706967 PMC5908901

[53] Trochez‐Martinez R, Smith P, Lamont R. Use of C‐reactive protein as a predictor of chorioamnionitis in preterm prelabour rupture of membranes: a systematic review. BJOG. 2007;114(7):796–801. doi: 10.1111/j.1471-0528.2007.01385.x 17567416

[54] Stepan M, Cobo T, Musilova I, Hornychova H, Jacobsson B, Kacerovsky M. Maternal Serum C-Reactive Protein in Women with Preterm Prelabor Rupture of Membranes. PloS one. 2016;11(3):e0150217. doi: 10.1371/journal.pone.0150217 . 26942752 PMC4778871

[55] Blanchong CA, Chung EK, Rupert KL, Yang Y, Yang Z, Zhou B, et al. Genetic, structural and functional diversities of human complement components C4A and C4B and their mouse homologues, Slp and C4. Int Immunopharmacol. 2001;1(3):365–92. doi: 10.1016/s1567-5769(01)00019-4 . 11367523

[56] Hoofnagle AN, Wener MH. The fundamental flaws of immunoassays and potential solutions using tandem mass spectrometry. J Immunol Methods. 2009;347(1–2):3–11. doi: 10.1016/j.jim.2009.06.003 . 19538965 PMC2720067

[57] Addona TA, Abbatiello SE, Schilling B, Skates SJ, Mani DR, Bunk DM, et al. Multi-site assessment of the precision and reproducibility of multiple reaction monitoring-based measurements of proteins in plasma. Nat Biotechnol. 2009;27(7):633–41. doi: 10.1038/nbt.1546 . 19561596 PMC2855883

[58] Kuzyk MA, Smith D, Yang J, Cross TJ, Jackson AM, Hardie DB, et al. Multiple reaction monitoring-based, multiplexed, absolute quantitation of 45 proteins in human plasma. Mol Cell Proteomics. 2009;8(8):1860–77. doi: 10.1074/mcp.M800540-MCP200 . 19411661 PMC2722777

[59] Wilson R. Sensitivity and specificity: twin goals of proteomics assays. Can they be combined? Expert Rev Proteomics. 2013;10(2):135–49. doi: 10.1586/epr.13.7 . 23573781

[60] Avula LR, Knapen D, Buckinx R, Vergauwen L, Adriaensen D, Van Nassauw L, et al. Whole-genome microarray analysis and functional characterization reveal distinct gene expression profiles and patterns in two mouse models of ileal inflammation. BMC Genomics. 2012;13:377. doi: 10.1186/1471-2164-13-377 . 22866923 PMC3599598

[61] Horvatic A, Guillemin N, Kaab H, McKeegan D, O’Reilly E, Bain M, et al. Quantitative proteomics using tandem mass tags in relation to the acute phase protein response in chicken challenged with Escherichia coli lipopolysaccharide endotoxin. J Proteomics. 2019;192:64–77. doi: 10.1016/j.jprot.2018.08.009 . 30114510

[62] Vajrychova M, Stranik J, Pimkova K, Barman M, Kukla R, Zednikova P, et al. Comprehensive proteomic investigation of infectious and inflammatory changes in late preterm prelabour rupture of membranes. Sci Rep. 2020;10(1):17696. doi: 10.1038/s41598-020-74756-9 . 33077789 PMC7573586

[63] Gomez-Lopez N, Romero R, Garcia-Flores V, Xu Y, Leng Y, Alhousseini A, et al. Amniotic fluid neutrophils can phagocytize bacteria: A mechanism for microbial killing in the amniotic cavity. Am J Reprod Immunol. 2017;78(4). doi: 10.1111/aji.12723 . 28703488 PMC5623137

[64] Merle NS, Noe R, Halbwachs-Mecarelli L, Fremeaux-Bacchi V, Roumenina LT. Complement System Part II: Role in Immunity. Front Immunol. 2015;6:257. doi: 10.3389/fimmu.2015.00257 . 26074922 PMC4443744

[65] Pan J, Tian X, Huang H, Zhong N. Proteomic Study of Fetal Membrane: Inflammation-Triggered Proteolysis of Extracellular Matrix May Present a Pathogenic Pathway for Spontaneous Preterm Birth. Front Physiol. 2020;11:800. doi: 10.3389/fphys.2020.00800 . 32792973 PMC7386131

[66] Parry S, Leite R, Esplin MS, Bukowski R, Zhang H, Varner M, et al. Cervicovaginal fluid proteomic analysis to identify potential biomarkers for preterm birth. Am J Obstet Gynecol. 2020;222(5):493 e1– e13. doi: 10.1016/j.ajog.2019.11.1252 . 31758918 PMC7196033

[67] Fortin T, Salvador A, Charrier JP, Lenz C, Lacoux X, Morla A, et al. Clinical quantitation of prostate-specific antigen biomarker in the low nanogram/milliliter range by conventional bore liquid chromatography-tandem mass spectrometry (multiple reaction monitoring) coupling and correlation with ELISA tests. Mol Cell Proteomics. 2009;8(5):1006–15. doi: 10.1074/mcp.M800238-MCP200 . 19068476 PMC2689759

[68] Valaperti A, Li Z, Vonow-Eisenring M, Probst-Muller E. Diagnostic methods for the measurement of human TNF-alpha in clinical laboratory. J Pharm Biomed Anal. 2020;179:113010. doi: 10.1016/j.jpba.2019.113010 . 31816469

[69] Blasco H, Lalmanach G, Godat E, Maurel MC, Canepa S, Belghazi M, et al. Evaluation of a peptide ELISA for the detection of rituximab in serum. J Immunol Methods. 2007;325(1–2):127–39. doi: 10.1016/j.jim.2007.06.011 . 17651747

[70] Geyer PE, Kulak NA, Pichler G, Holdt LM, Teupser D, Mann M. Plasma Proteome Profiling to Assess Human Health and Disease. Cell Syst. 2016;2(3):185–95. doi: 10.1016/j.cels.2016.02.015 . 27135364

[71] Bellei E, Bergamini S, Monari E, Fantoni LI, Cuoghi A, Ozben T, et al. High-abundance proteins depletion for serum proteomic analysis: concomitant removal of non-targeted proteins. Amino Acids. 2011;40(1):145–56. doi: 10.1007/s00726-010-0628-x . 20495836

[72] Tu C, Rudnick PA, Martinez MY, Cheek KL, Stein SE, Slebos RJ, et al. Depletion of abundant plasma proteins and limitations of plasma proteomics. J Proteome Res. 2010;9(10):4982–91. doi: 10.1021/pr100646w . 20677825 PMC2948641

[73] Fisk NM, Fysh J, Child AG, Gatenby PA, Jeffery H, Bradfield AH. Is C-reactive protein really useful in preterm premature rupture of the membranes? Br J Obstet Gynaecol. 1987;94(12):1159–64. doi: 10.1111/j.1471-0528.1987.tb02316.x . 3426987

[74] Murtha AP, Sinclair T, Hauser ER, Swamy GK, Herbert WN, Heine RP. Maternal serum cytokines in preterm premature rupture of membranes. Obstet Gynecol. 2007;109(1):121–7. doi: 10.1097/01.AOG.0000250474.35369.12 . 17197597

[75] Kwak HM, Shin MY, Cha HH, Choi SJ, Lee JH, Kim JS, et al. The efficacy of cefazolin plus macrolide (erythromycin or clarithromycin) versus cefazolin alone in neonatal morbidity and placental inflammation for women with preterm premature rupture of membranes. Placenta. 2013;34(4):346–52. doi: 10.1016/j.placenta.2013.01.016 . 23465535

[76] Lee J, Romero R, Kim SM, Chaemsaithong P, Park CW, Park JS, et al. A new anti-microbial combination prolongs the latency period, reduces acute histologic chorioamnionitis as well as funisitis, and improves neonatal outcomes in preterm PROM. J Matern Fetal Neonatal Med. 2016;29(5):707–20. doi: 10.3109/14767058.2015.1020293 . 26373262 PMC5704947

[77] Kacerovsky M, Romero R, Stepan M, Stranik J, Maly J, Pliskova L, et al. Antibiotic administration reduces the rate of intraamniotic inflammation in preterm prelabor rupture of the membranes. Am J Obstet Gynecol. 2020;223(1):114 e1–e20. doi: 10.1016/j.ajog.2020.01.043 . 32591087 PMC9125527

