# Supplementary figures and images for: Plasma proteomic analysis to identify potential biomarkers of histologic chorioamnionitis in women with preterm premature rupture of membranes
Source: PLoS One. 2022 Jul 7;17(7):e0270884. doi: 10.1371/journal.pone.0270884 (PMC9262229; doi:10.1371/journal.pone.0270884)

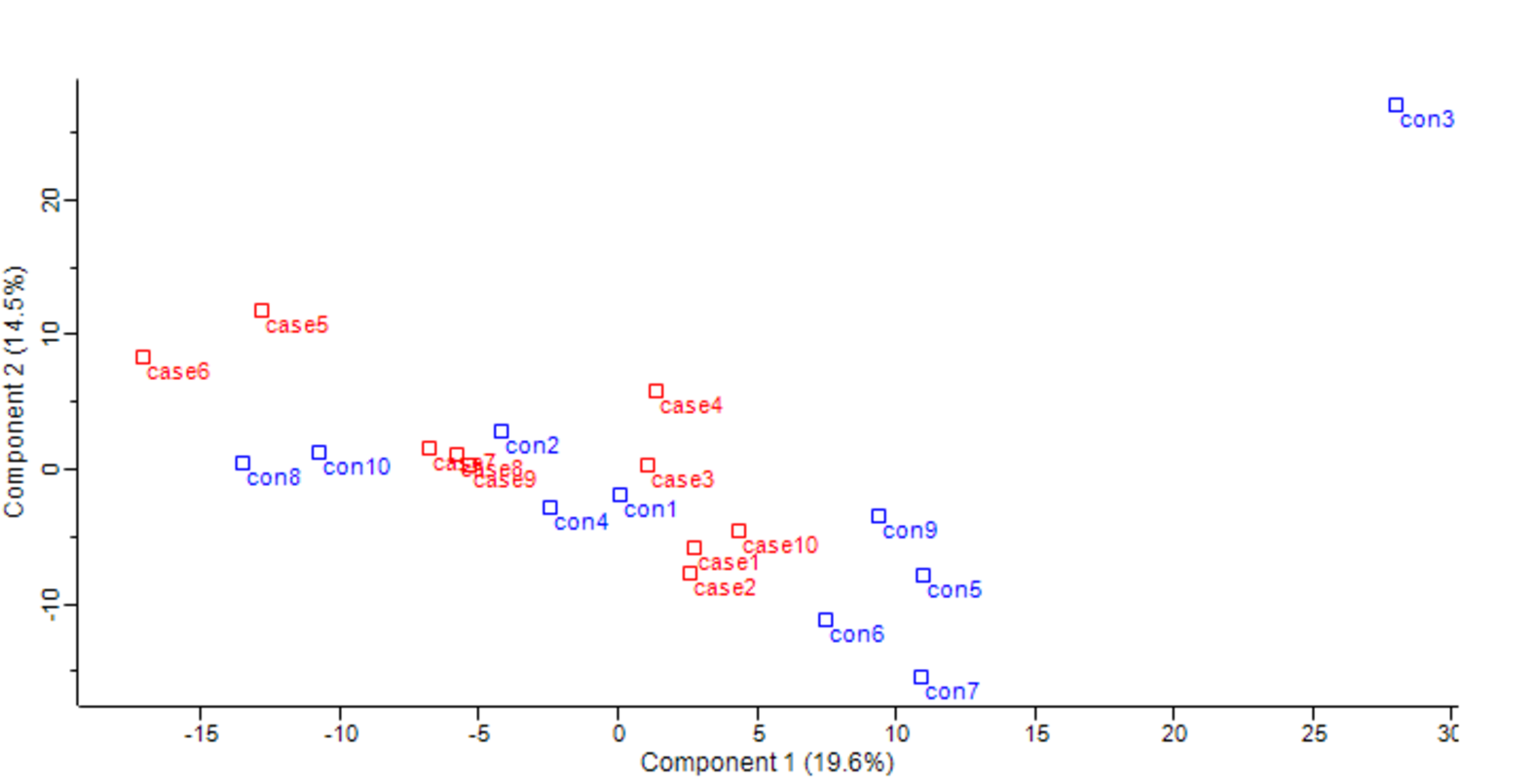

Supplement: S1 Fig — (TIF) [file pone.0270884.s008.tif]

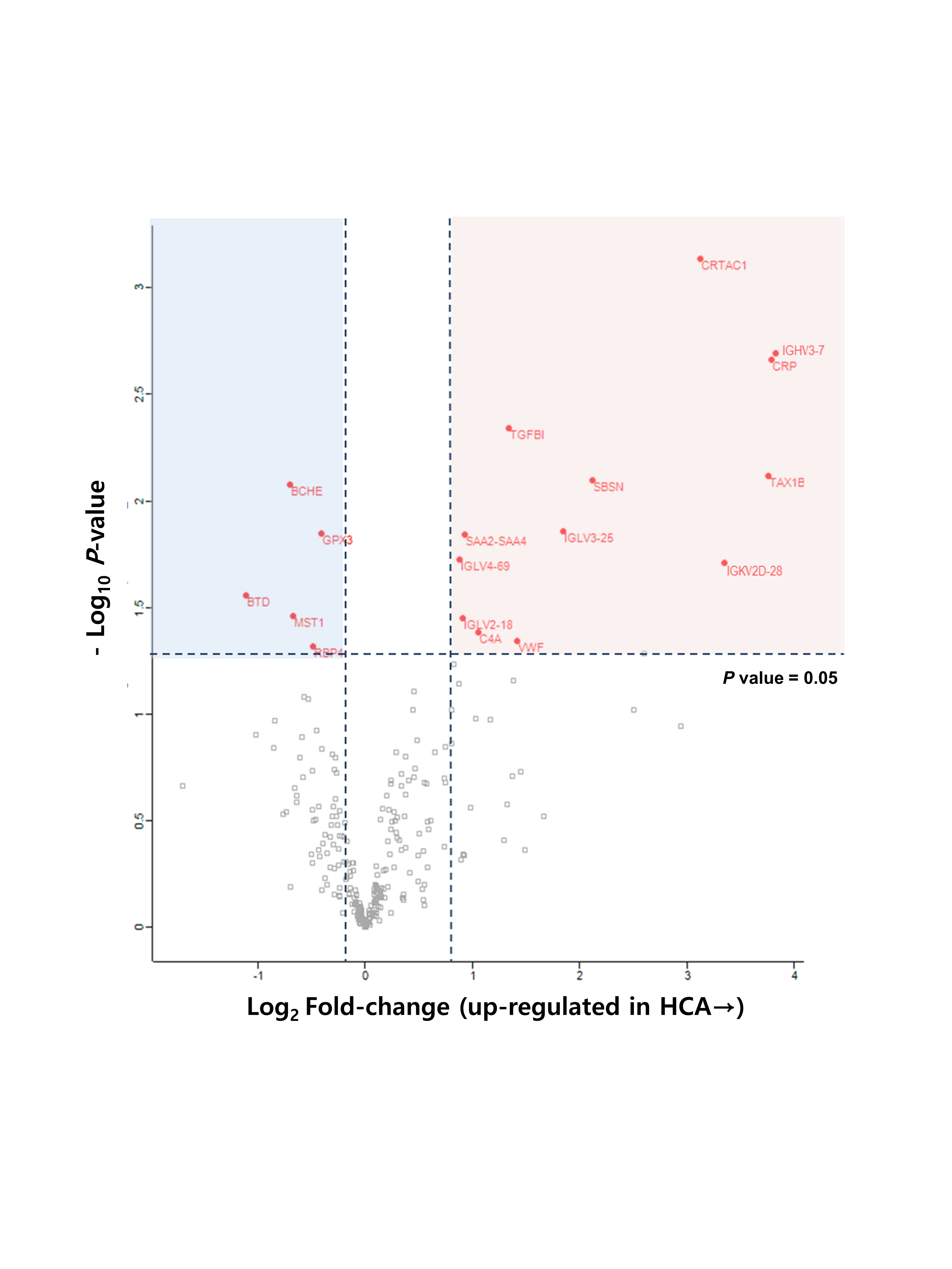

Supplement: S2 Fig — Volcano plot shows the plasma upregulated and downregulated differentially expressed proteins (DEPs) in the histologic chorioamnionitis (HCA) and non-HCA groups. Representative protein identifiers in red indicate statistically significant DEPs (P < 0.05). (TIF) [file pone.0270884.s009.tif]

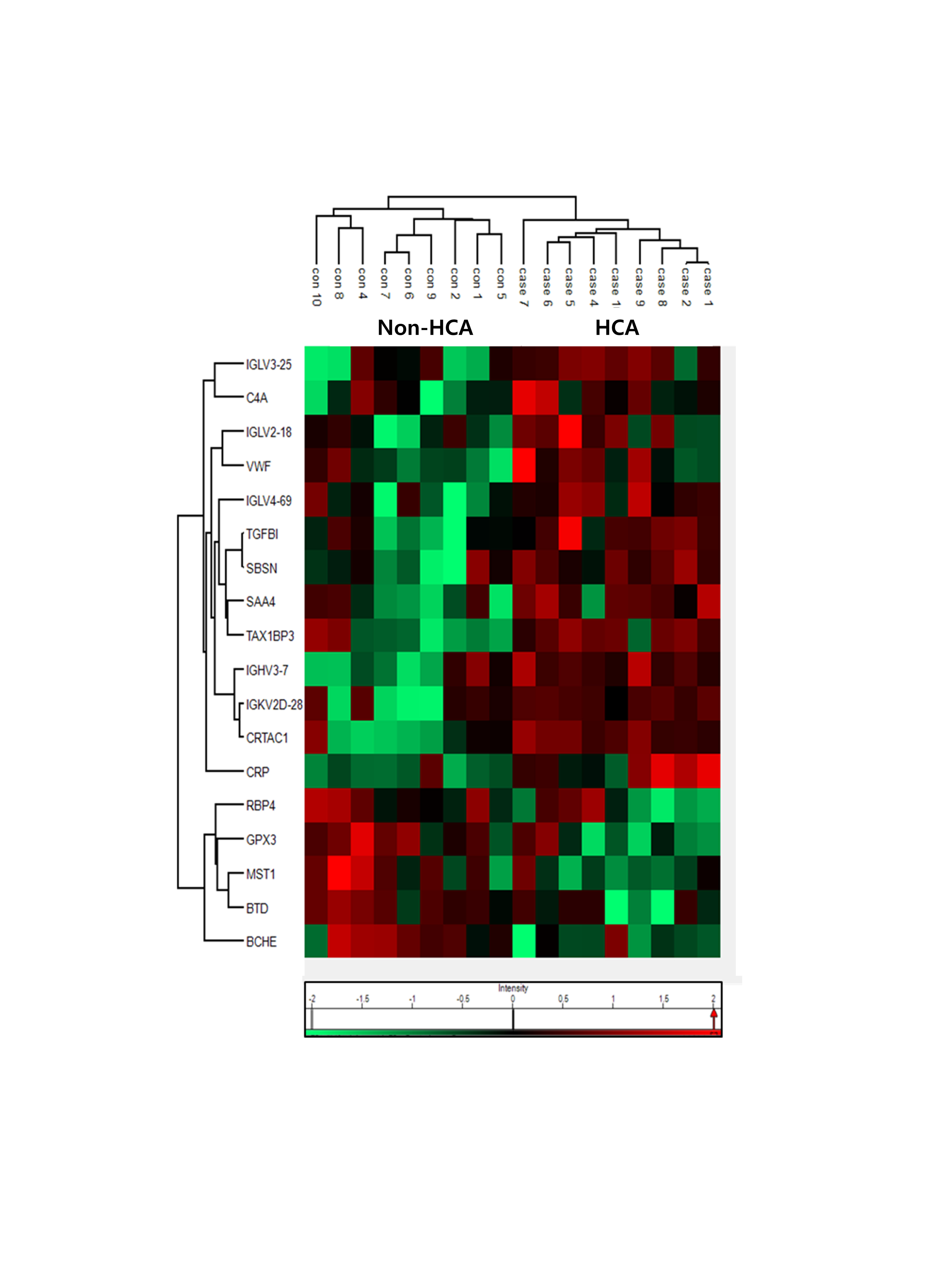

Supplement: S3 Fig — (TIF) [file pone.0270884.s010.tif]

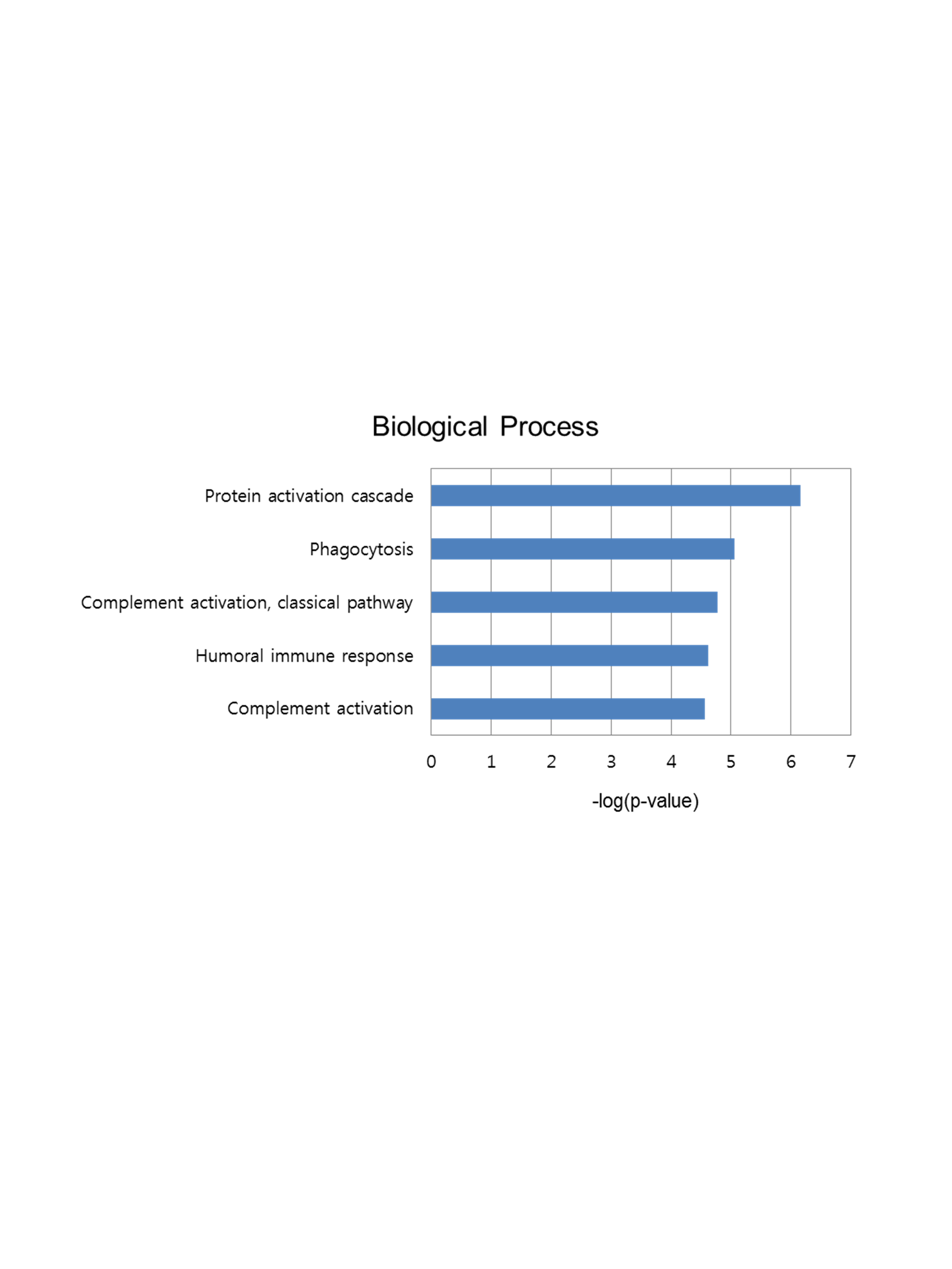

Supplement: S4 Fig — (TIF) [file pone.0270884.s011.tif]

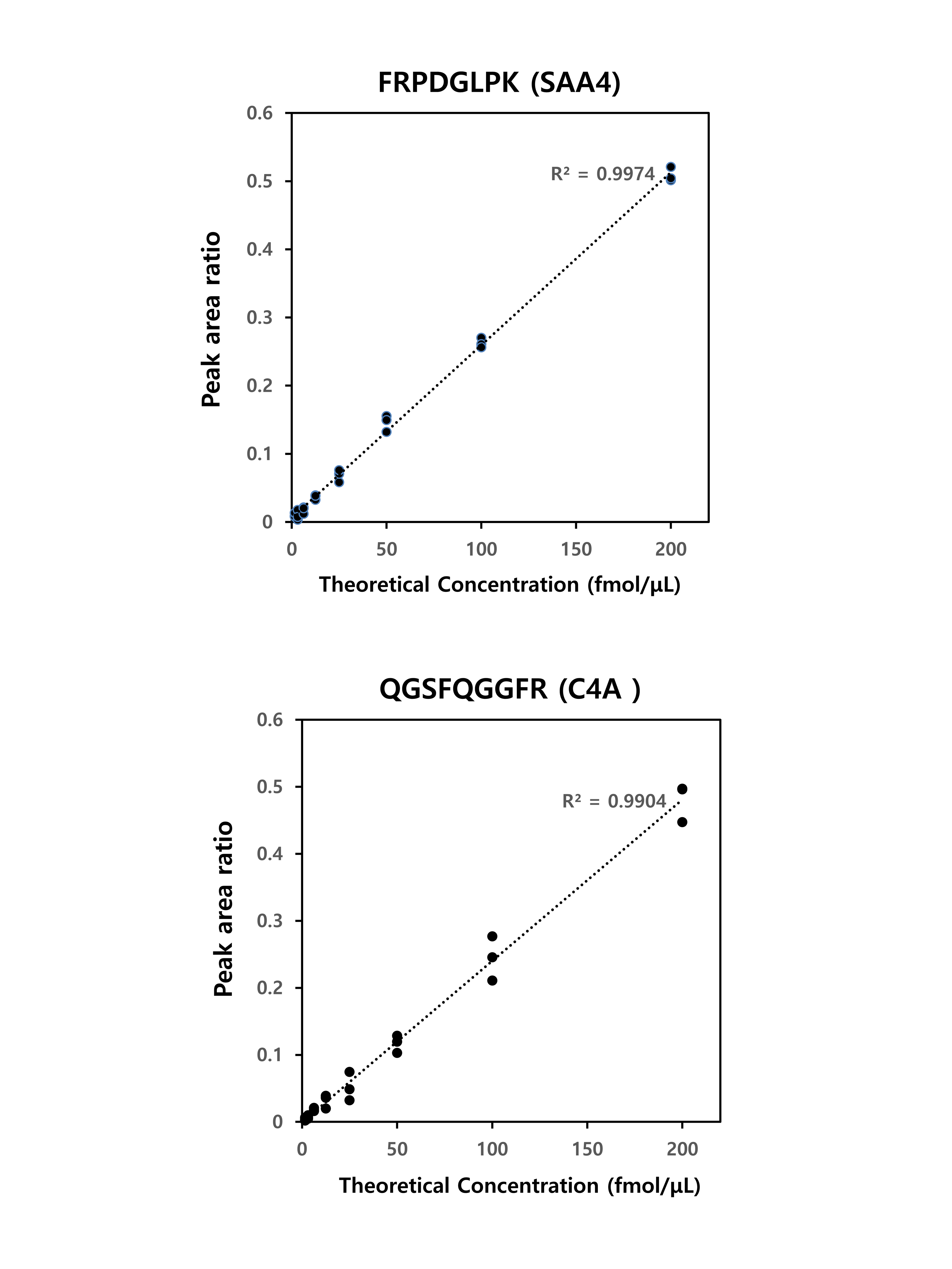

Supplement: S5 Fig — The peptides correspond to FRPDGLPK and QGSFQGGFR from SAA4 and C4A, respectively. For each concentration point, triplicates were analyzed. C4A, complement C4-A; SAA4, serum amyloid A4. (TIF) [file pone.0270884.s012.tif]

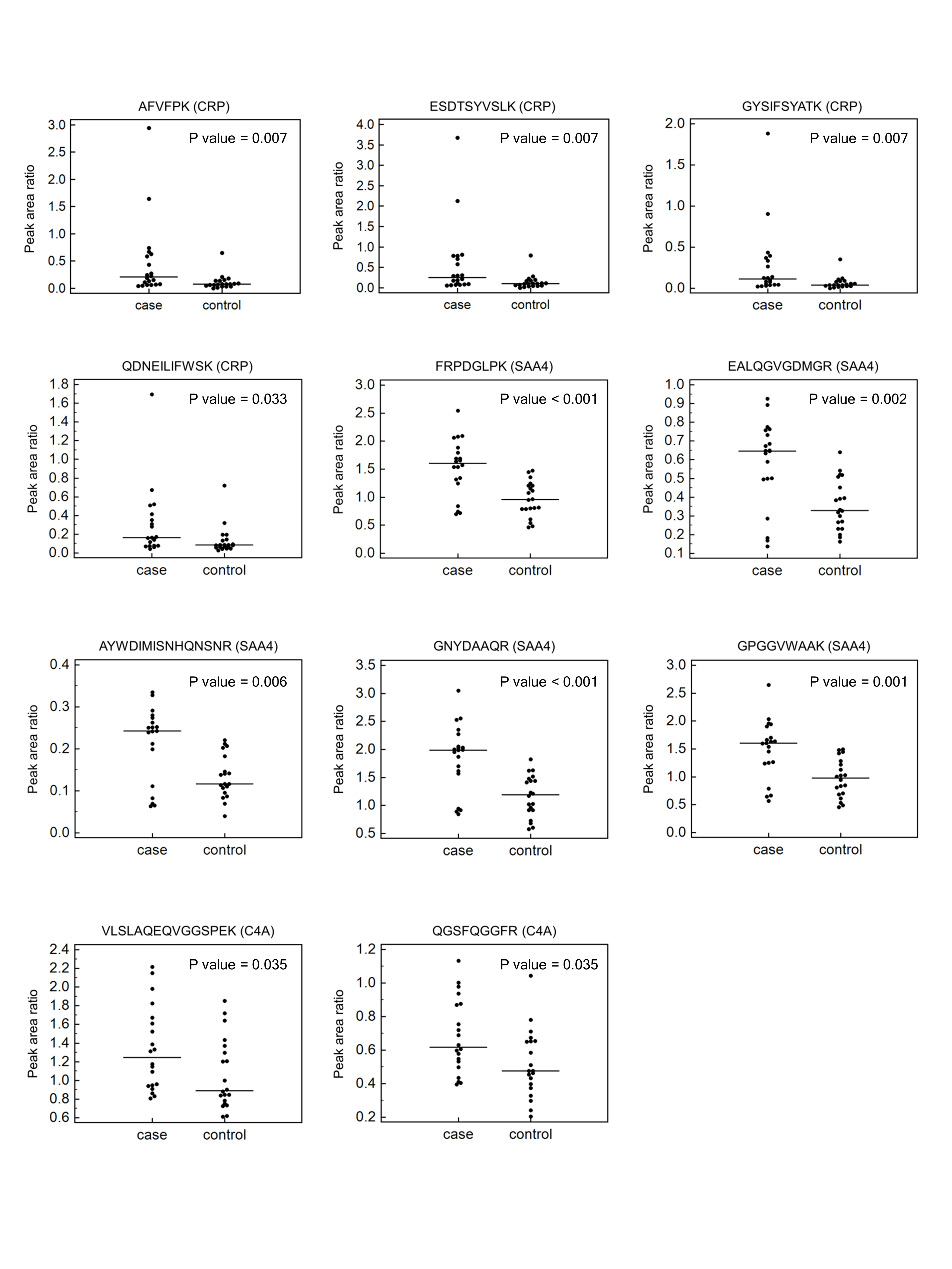

Supplement: S6 Fig — Interactive plots were generated using the normalized peak area of each MRM target peptide. The median levels of CRP, C4A, and SAA4 in plasma were significantly higher in women with HCA than in the non-HCA control group. The horizontal line in each figure represents the median value. C4A, complement C4-A; CRP, C-reactive protein; HCA, histologic chorioamnionitis; PPROM, preterm premature rupture of membranes; SAA4, serum amyloid A4. (TIF) [file pone.0270884.s013.tif]
