# Supplementary material for: Plasma proteomic analysis to identify potential biomarkers of histologic chorioamnionitis in women with preterm premature rupture of membranes
Source: PLoS One. 2022 Jul 7;17(7):e0270884. doi: 10.1371/journal.pone.0270884 (PMC9262229; doi:10.1371/journal.pone.0270884)
Supplement: S1 Text — (DOCX) [file pone.0270884.s018.docx]

## - Supplementary Materials -

**The methods for placental tissue collection and processing for histologic evaluation**

Tissue samples of the placenta were obtained from the placental membranes, umbilical cord, and chorionic plate. They were fixed in 10% neutral-buffered formalin and embedded in paraffin. Sections of the tissue blocks were stained with hemotoxylin and eosin. Histopathologic examination was performed by experienced board-certified pathologists who were blinded to the clinical information. The presence of acute inflammation was noted and classified as grade 1 or 2 according to criteria previously published.^1^

**Management of preterm premature rupture of membranes** **(PPROM)**

Prophylactic broad-spectrum antibiotics [ampicillin plus macrolides (azithromycin, clarithromycin or erythromycin)] were administered in all women with PPROM. Antenatal corticosteroids were administered to mature fetal lungs when PPROM occurred between 24.0 and 34.0 weeks of gestation. Tocolytic therapy (magnesium sulfate, ritodrine, or atosiban) was administered in women with PPROM less than 34 weeks at the discretion of the attending obstetrician. Maternal or fetal health status was carefully monitored for the development of clinical sign of chorioamnionitis and/or fetal compromise, both of which are indications for induction of labor. In most patients with culture-proven microbial invasion of amniotic cavity (MIAC), labor was not induced or an elective cesarean delivery was not performed purely for positive amniotic fluid (AF) cultures. In women with a diagnosis of MIAC (i.e., positive AF cultures) but without clinical chorioamnionitis, the effective antibiotics against isolated bacteria were administered and close monitoring for clinical sign of chorioamnionitis and/or fetal compromise was performed until 34 weeks of gestation. Induction of labor was performed after 34 weeks if clinical sign of chorioamnionitis, fetal compromise or labor had not developed. Acute histologic chorioamnionitis was diagnosed when acute inflammatory change was detected in any tissue sample (umbilical cord, chorionic plate, chorion-decidua, or amnion), in accordance with previously published criteria.^1^ Clinical chorioamnionitis was diagnosed following the criteria proposed by Gibbs et al.^2^; fever (≥37.8°C) and the presence of two or more of the associated clinical findings (uterine tenderness, malodorous vaginal discharge, maternal leukocytosis, maternal tachycardia, and fetal tachycardia).

**Plasma sample preparation**

The protein digestion procedure was performed with 2 **μL** of each plasma sample as previously described.^3^ Briefly, 23 **μL** of digestion buffer including denaturation, reduction and alkylation reagents (8 M urea, 5 mM tris(2-carboxyethyl)phosphine, 20 mM iodoacetamide (IAA) in 0.1 M ammonium bicarbonate) were added to 2 μL of blood plasma. The mixture was boiled for 25 min at 60℃ to denature, reduce, and alkylate the proteins. After boiling the proteins, samples were cooled down to room temperature. Then protein digestion was performed at 37℃ overnight using a trypsin at a 100:1 proteins-to-protease ratio. After that process, all peptides were acidified with 10% of trifluoroacetic acid (TFA), then desalted using homemade C18-StageTips method as described.^4^ The Desalted peptides were vacuum-centrifuged for drying and the stored at -80℃.

**Liquid chromatography-tandem mass spectrometry (LC-MS/MS) analysis (Discovery phase)**

LC-MS/MS analysis was performed using Quadrupole Orbitrap mass spectrometers, Q-Exactive plus (Thermo Fisher Scientific, Waltham, MA, USA) coupled to an Ultimate 3000 RSLC systems (Dionex, Sunnyvale, CA, USA) with a nano electrospray source. Prior to sample injection, the dried peptide samples were reconstituted in solvent A (0.1% formic acid in 2% acetonitrile [ACN]). Peptides were then injected and trapped on trapping column (75 µm I.D. × 2 cm, C18, 3 µm, 100 Å) for removal of salts and separated on analytical column (50 µm I.D. × 15 cm, C18, 1.9 µm, 100 Å). After the samples were loaded onto the nano LC, a 90-min gradient from 6% to 30% solvent B (100% ACN and 0.1% formic acid) was applied to all samples. The spray voltage was 2.0 kV in positive ion mode and the temperature of the heated capillary was set to 320°C. Mass spectra were acquired in data-dependent mode using a top 15 method on a Q Exactive Plus. The Orbitrap analyzer scanned precursor ions with a mass range of 300–1650 m/z and resolution of 70,000 at m/z 200. Higher-energy collisional dissociation (HCD) scans were acquired on the Q Exactive at a resolution of 17,500. HCD peptide fragments were acquired at a normalized collision energy of 28. The maximum ion injection times for the survey and MS/MS scans were 20 and 120 msec, respectively.

**Data processing for label-free quantification**

Mass spectra were processed in MaxQuant version 1.6.1.0.^5, 6^ MS/MS spectra were searched against the Human UniProt protein sequence database (release date: December 2014) using the Andromeda search engine.^7^ Primary searches were performed using a 6-ppm precursor ion tolerance for total protein level analysis. The MS/MS ion tolerance was set to 20 ppm. Cysteine carbamido-methylation was set as a fixed modification. N-Acetylation of proteins and oxidation of methionine were set as variable modifications. Enzyme specificity was set to full tryptic digestion. Peptides with a minimum length of six amino acids and up to two missed cleavages were considered. The required false discovery rate (FDR) was set to 1% at the peptide, protein, and modification levels. All proteins were identified by one or more unique peptides. For label-free quantification of each protein in the plasma was estimated by determining their Intensity Based Absolute Quantification (iBAQ, the sum of all of the identified peptides peak intensities divided by the number of theoretically observable tryptic peptides) values which is embedded algorithm in MaxQuant software.^8^ To maximize the number of quantification events across samples, we enabled the ‘Match between Runs’ options on the MaxQuant.

**Statistical analysis (Discovery)**

Statistical analyses for the identified and label-free quantified protein data were conducted using Perseus software.^9^ Initially, proteins identified as only identified by site, reverse, and contaminants were removed. IBAQ intensity of each protein was used for comparison of expression level between groups. Because of the skewed distribution of the data, iBAQ intensity of each protein was log2 transformed. Valid values were filtered with proteins with a minimum of 60% quantified values in at least one group. In total, 7.2% of data values (350 of 4860) were missing and imputed based on a normal distribution (width = 0.3, down-shift = 1.8) to simulate signals of low-abundance proteins. Two-sided Student’s t-tests were performed for pairwise comparison of proteomes to detect differentially expressed proteins (DEPs). DEPs (*P* < 0.05) were selected as statistically significant. The protein abundances were subjected to z-normalization followed by hierarchical clustering with Euclidean distance. Over representation analysis of 18 DEPs using DAVID gene ontology (<http://david.abcc.ncifcrif.gov/>) was done according to biological process, cellular component, and molecular function terms. Statistical significance of the enriched gene ontology term was determined by the Fisher’s exact test. All statistical tests were two-sided, and *P* < 0.05 was considered as statistically significant.

**Plasma sample preparation for quantification of target peptides with LC-multiple reaction monitoring (MRM)-MS**

Eighteen microliters of each plasma sample diluted with 72 μL of buffer A (Agilent) was passed through 0.22 μm spin filters (Agilent). From supernatant, six highly abundant human plasma proteins (albumin, immunoglobulin A, immunoglobulin G, serotransferrin, haptoglobin, and alpha-1 antitrypsin) were then removed using a MARS column (Hu-6HC, Agilent). An Infinity 1260 Bioinert high-performance liquid chromatography (HPLC) system with a fraction collector was used to collect flow-through fractions. Depleted plasma was concentrated by centrifugal filtration using the 3 kDa molecular weight cut-off filter (Amicon**^®^** Ultra 3K, Millipore). The concentrated plasma protein was quantified by BCA^TM^ Protein assay kit (Pierce^®^, USA).

Equal amounts of protein (100 μg) from each sample were denatured with 0.1% (final concentration) RapiGest^TM^ surfactant (Waters, Milford, USA) for 20 min at 80°C. Denatured proteins were reduced by the addition of 50 mM dithiothreitol (Merck, Darmstadt, Germany) in 50 mM ammonium bicarbonate, followed by incubation at 60°C for 30 min. The proteins were then alkylated by the addition of 150 mM IAA (Sigma, St. Louis, MO, USA), followed by incubation at room temperature for 30 min in the dark. Sequencing-grade modified trypsin was added to samples at a 100:1 ratio (plasma proteins substrate: trypsin ratio [w/w]). Trypsin digestion was performed for 16 h at 37°C. Tryptic digestion was quenched by the addition of 1% TFA at a final concentration of 0.5 % (v/v), followed by incubation for 45 min at 37°C. The peptides generated from trypsin digestion were desalted using OASIS HLB 1-cc (30 mg) desalting cartridges (Waters). The desalted peptides were lyophilized on a speed vacuum centrifuge (CcntriVap**^®^** centrifugal concentrator, LABCONCO, Kansas City, MO, USA) and stored at -80°C until use. Prior to LC-MRM-MS analysis, the samples were reconstituted in 25 **μL** of 0.1% formic acid in 2% ACN and mixed with mixtures of stable heavy isotope-labeled peptides. Samples were then centrifuged at 18,000 g for 12 minutes, 6 **μL** of samples were injected for LC-MRM MS analysis. The concentrations of spiked heavy peptides were adjusted to the peak areas of the corresponding endogenous peptides so that the light (endogenous peptide) to heavy peptide peak area ratios ranged from 0.1 to 10.

**LC-MRM-MS analysis**

MRM analysis was performed on a 6495 Triple-Quadrupole Mass Spectrometer coupled to an Agilent 1260 Infinity HPLC system (Agilent). Twenty micrograms of digested peptides sample was loaded onto a reversed-phase analytical column (Agilent ZORBAX SB-C18, 3.5 µm, 15 cm in length, 500 µm inner diameter). The temperature of the analytical column was maintained at 40°C. Peptide separation was performed at a flow rate of 18 μL/min on a linear gradient of mobile phase B (0.1% formic acid in ACN) from 2 % to 35 % over 35 min. The total run time was 60 min (mobile phase A: 0.1% formic acid in water, mobile phase B: 0.1% formic acid in ACN). A triple quadrupole LC/MS system (6495 LCMS, Agilent) was used for the detection of m/z and the signal intensity of peptides eluted from the analytical column. Automated optimization of the electrospray source parameters and mass axis calibration in ESI positive mode were performed with tuning solution (G1969-85000, Agilent), using the autotune function in MassHunter Workstation software (ver B.08.00, Agilent). The drying gas temperature was set at 250°C at a flow rate of 15 L/min, while the sheath gas temperature was set at 300°C at a flow rate of 12 L/min. The nebulizer gas flow was set at 30 psi. MRM-MS analysis was performed in the positive ion mode, with the ion spray capillary voltage and nozzle voltage set at 3,100 and 1,200 V, respectively. The delta electron multiplier voltage was set at 300 V and the cell accelerator voltage and fragment voltages were set at 5 V and 380 V, respectively. The resolution of MS quadrupole-1 and quadrupole-3 was set to unit resolution (0.7 full width at half maximum).

**Optimization of the multiplexed MRM-MS method for the quantification of target proteins using heavy isotope-labeled peptides**

Target peptides and transition lists for the optimization of MRM method were generated from 12 DEPs extracted from label-free based discovery results using Skyline software (ver 4.2, University of Washington, Seattle, WA, USA).^10^ The appropriate selection of surrogate peptides for MRM method development was conducted according to the following criteria: 1) peptides identified from discovery experiments or present in a plasma proteome MS/MS library constructed in-house; 2) peptides present in the database ([http://www.srmatlas.org](http://www.srmatlas.org/)) or MRMAssayDB (<http://mrmassaydb.proteincentre.com).>^11^ Peptides met at least one criterion were selected. Finally, 49 heavy isotope-labeled peptide standards (SpikeTides^TM^; JPT Peptide Technologies, Berlin, Germany) were synthesized and evaluated for suitability.

To evaluate the suitability of the heavy peptides for LC-MRM-MS method optimization, we identified the retention time, optimized collisional energy values, and MS signal intensity for heavy peptides by spiking them into a tryptic peptide matrix obtained from the pooled plasma samples used for discovery experiments. All raw MRM-MS data were processed using Skyline software. All integrated peaks were manually inspected to confirm correct peak detection and accurate integration. The MRM acquisition method was initially built with eight to fourteen precursor/fragment ion pairs (transitions) for each light and heavy peptide. Each light and heavy transition was carefully reviewed to refine the transitions showing a better MS response, no interference, and consistency of signal intensity over consecutive LC-MS runs. We selected 3-4 MRM transitions with the best intensity and linearity for each surrogate peptide, refining the MRM transition process.

Reverse response curves for examining linearity were generated from MRM-MS data from each serially diluted heavy peptide spiked into 25 μg of trypsin-digested plasma proteins. Eight concentration points (approximately 0.975–500 fmol/µL) were used, and a trypsin-digested pooled plasma protein without heavy peptides was used as a blank to estimate background levels. Triplicate MRM-MS runs were performed at each concentration point. Plots of the peak area ratio of heavy to light peptides versus the heavy peptide area of eight concentration points were generated and linearity and coefficients of variation (CVs) were evaluated using QUASAR software (external software installed in SKYLINE). MRM transitions of each heavy peptide with a CV < 20% and a coefficient of determination > 0.98 were selected as refined transitions for quantification of the corresponding endogenous peptides. Representative response curves of the heavy peptides are shown in S5 Fig. The target cycle time for the multiplexed MRM-MS method was set at 1,500 msec. Dwell times were adjusted automatically depending on the number of concurrent transitions. Minimum dwell time was set at 10 msec. The retention time tolerance window for scheduled MRM-MS methods was 3.3 min. Finally, we established a multiplexed MRM method that could quantify 49 surrogate peptides derived from 12 proteins in one LC-MS injection. The optimized MRM parameters for 334 transitions for 49 peptides are shown in Table S5. Using this MRM MS method, for relative quantitation of target peptides were performed in 40 individual plasma samples. Two technical LC-MRM-MS replicate runs were performed for each sample. Peak area ratio (PAR) of each MRM transition was measured and processed by Skyline software for relative quantitation of the target peptides of 40 individual plasma samples. Averaged PAR value for each peptide was used for further statistical analysis.

**Analysis of complement C4-A (C4A) and serum amyloid A4 protein (SAA4) in the plasma samples during validation phase**

The ranges of C4A and SAA4 standard curves were 31.2-2000 pg/mL and 78.1-5000 pg/mL, respectively. Prior to measurement of these proteins, the maternal plasma samples were diluted at 1:4 for SAA4 and 1:100000 for C4A. The intra- and inter-assay coefficients of variation were 4.1% and 6.6% for C4A and 4.1% and 10.2% for SAA4, respectively.

**For further details on the inclusion of study participants employed for proteomic studies in the discovery and verification stages**

The patients were initially divided into two groups at the discovery stage, comprising 10 cases and 10 controls. PCA was performed using the whole data set, and a single outlier was identified by visual inspection of a scatterplot (Fig S1). The amount of peptides in this outlier plasma sample was significantly lower than that in other samples. The amount of peptides in the outlier ranged from 23.3% to 36.8% compared to that in the remaining samples. Hence, this outlier sample (control 3) and one case sample (case 3) matched with control 3 were excluded from the discovery phase of the study. The final analysis was performed using 9 cases and 9 controls. This outlier sample was also excluded from further analyses performed during the verification and validation phases. Instead, a new control sample that could replace control 3, which was excluded as an outlier, was reassigned to the control group in the verification cohort. The sample was included on the basis of the gestational age at sampling from the remaining samples for the validation phase. One case sample (case 3) that was matched with control 3 was still included in the analysis of the verification phase.

**Reference**

1. Jung EY, Choi BY, Rhee J, Park J, Cho SH, Park KH. [Relation between amniotic fluid infection or cytokine levels and hearing screen failure in infants at 32 wk gestation or less.](https://www.ncbi.nlm.nih.gov/pubmed/27925622) Pediatr Res 2017;81(2):349-355.
2. Gibbs, R. S., Blanco, J. D., St Clair, P. J. & Castaneda, Y. S. Quantitative bacteriology of amniotic fluid from women with clinical intraamniotic infection at term. The Journal of infectious diseases 1982;145:1-8.
3. Rhee, S.J., Han, D., Lee, Y. et al. Comparison of serum protein profiles between major depressive disorder and bipolar disorder. BMC Psychiatry 20, 145 (2020).
4. Han D, Jin J, Woo J, Min H, Kim Y. Proteomic analysis of mouse astrocytes and their secretome by a combination of FASP and StageTip-based, high pH, reversed-phase fractionation. Proteomics. 2014;14(13-14):1604-9.
5. Cox J, Mann M. MaxQuant enables high peptide identification rates, individualized p.p.b.-range mass accuracies and proteome-wide protein quantification. Nat Biotechnol. 2008;26(12):1367-72. doi: 10.1038/nbt.1511. PubMed PMID: 19029910.
6. Tyanova S, Temu T, Cox J. The MaxQuant computational platform for mass spectrometry-based shotgun proteomics. Nat Protoc. 2016;11(12):2301-19. doi: 10.1038/nprot.2016.136. PubMed PMID: 27809316.
7. Cox J, Neuhauser N, Michalski A, Scheltema RA, Olsen JV, Mann M. Andromeda: a peptide search engine integrated into the MaxQuant environment. J Proteome Res. 2011;10(4):1794-805.
8. Schwanhäusser B, Busse D, Li N, Dittmar G, Schuchhardt J, Wolf J, Chen W, Selbach M. Corrigendum: Global quantification of mammalian gene expression control. Nature. 2013;495(7439):126-7.
9. Tyanova S, Temu T, Sinitcyn P, Carlson A, Hein MY, Geiger T, Mann M, Cox J. The Perseus computational platform for comprehensive analysis of (prote)omics data. Nat Methods. 2016;13(9):731-40.
10. MacLean B, Tomazela DM, Shulman N, Chambers M, Finney GL, Frewen B, Kern R, Tabb DL, Liebler DC, MacCoss MJ. Skyline: an open source document editor for creating and analyzing targeted proteomics experiments. Bioinformatics. 2010;1;26(7):966-8.
11. Bhowmick P, Mohammed Y, Borchers CH. MRMAssayDB: an integrated resource for validated targeted proteomics assays. Bioinformatics. 2018;34(20):3566-3571.
